# Safety and biomarker effects of candesartan in non-hypertensive adults with prodromal Alzheimer’s disease

**DOI:** 10.1093/braincomms/fcac270

**Published:** 2022-10-25

**Authors:** Ihab Hajjar, Maureen Okafor, Limeng Wan, Zhiyi Yang, Jonathon A Nye, Anastasia Bohsali, Leslie M Shaw, Allan I Levey, James J Lah, Vince D Calhoun, Reneé H Moore, Felicia C Goldstein

**Affiliations:** Department of Neurology, Emory University School of Medicine, Atlanta, GA 30329, USA; Department of Neurology, University of Texas Southwestern, Dallas, TX 75390, USA; Department of Neurology, Emory University School of Medicine, Atlanta, GA 30329, USA; Department of Biostatistics and Bioinformatics, Emory University Rollins School of Public Health, Atlanta, GA 30322, USA; Department of Neurology, Emory University School of Medicine, Atlanta, GA 30329, USA; Department of Radiology and Imaging Sciences, Center for Systems Imaging, Emory University, Atlanta, GA 30329, USA; Tri-institutional Center for Translational Research in Neuroimaging and Data Science, Georgia State University, Georgia Institute of Technology, Emory University, Atlanta, GA 30303, USA; Department of Pathology and Laboratory Medicine and Center for Neurodegenerative Disease Research, University of Pennsylvania, PA 19104, USA; Department of Neurology, Emory University School of Medicine, Atlanta, GA 30329, USA; Department of Neurology, Emory University School of Medicine, Atlanta, GA 30329, USA; Tri-institutional Center for Translational Research in Neuroimaging and Data Science, Georgia State University, Georgia Institute of Technology, Emory University, Atlanta, GA 30303, USA; Department of Biostatistics and Bioinformatics, Emory University Rollins School of Public Health, Atlanta, GA 30322, USA; Department of Neurology, Emory University School of Medicine, Atlanta, GA 30329, USA

**Keywords:** candesartan, angiotensin receptor blockade, prodromal Alzheimer’s disease, cognition, mild cognitive impairment

## Abstract

Observational studies suggest that angiotensin receptor blockers in hypertensive adults are associated with lower post-mortem indicators of Alzheimer’s disease pathology. Candesartan, an angiotensin receptor blocker, has a positive cognitive effect in mild cognitive impairment with hypertension. However, its safety and effects in non-hypertensive individuals with Alzheimer’s disease are unclear. This is the first double-blind randomized placebo-controlled trial aimed to assess safety and effects of 1-year therapy of candesartan on biomarkers and clinical indicators of Alzheimer’s disease in non-hypertensive individuals with biomarker-confirmed prodromal Alzheimer’s disease. Seventy-seven non-hypertensive participants 50 years or older (mean age: 68.1 years; 62% women; 20% African American) with mild cognitive impairment and biomarker confirmed Alzheimer’s disease were randomized to escalating doses of once daily oral candesartan (up to 32 mg) or matched placebo. Main outcomes included safety and tolerability of candesartan, cerebrospinal fluid biomarkers (amyloid-β42, amyloid-β40, total tau and phospho-tau). Additional exploratory outcomes included PET imaging (Pittsburgh Compound-B (^11^C-PiB) and ^18^F-flortaucipir), brain MRI (structural and connectivity measures) and cognitive functioning. Analyses used intention-to-treat approach with group comparisons of safety measures using Chi-square test, and repeated measures mixed effects models were used to assess candesartan effects on main and exploratory outcomes (ClinicalTrials.gov, NCT02646982). Candesartan was found to be safe with no significant difference in safety measures: symptoms of hypotension, renal failure or hyperkalemia. Candesartan was also found to be associated with increases in cerebrospinal fluid Aβ40 (between-group mean difference: 1211.95 pg/ml, 95% confidence interval: 313.27, 2110.63) and Aβ42 (49.51 pg/ml, 95% confidence interval: −98.05, −0.98) reflecting lower brain amyloid accumulation. Candesartan was associated with decreased ^11^C-PiB in the parahippocampal region (−0.1104, 95% confidence interval: −0.19, −0.029) which remained significant after false discovery rate correction, and with an increase in functional network connectivity in the subcortical networks. Candesartan was further associated with improved executive function (Trail Making Test Part B) performance (−11.41 s, 95% confidence interval: −11.94, −10.89) and trended for an improved global cognitive functioning reflected by a composite cognitive score (0.002, 95% confidence interval: −0.0002, 0.005). We did not observe significant effects on tau levels, hippocampal volume or other cognitive measures (memory or clinical dementia rating scale-sum of boxes). In conclusion, among non-hypertensive prodromal Alzheimer’s disease, candesartan is safe and likely decreases brain amyloid biomarkers, enhances subcortical brain connectivity and has favourable cognitive effects. These findings suggest that candesartan may have an important therapeutic role in Alzheimer’s disease, and warrant further investigation given the lack of clear treatment options for this devastating illness.

## Teaser

Hajjar et al report that candesartan treatment may provide significant favorable effects on brain amyloid accumulation and executive function in non-hypertensives with prodromal Alzheimer’s disease. This offers new evidence of its amyloid cascade engagement and its potential role in Alzheimer's disease-targeted therapy.

## Graphical Abstract

**Graphical abstract:**
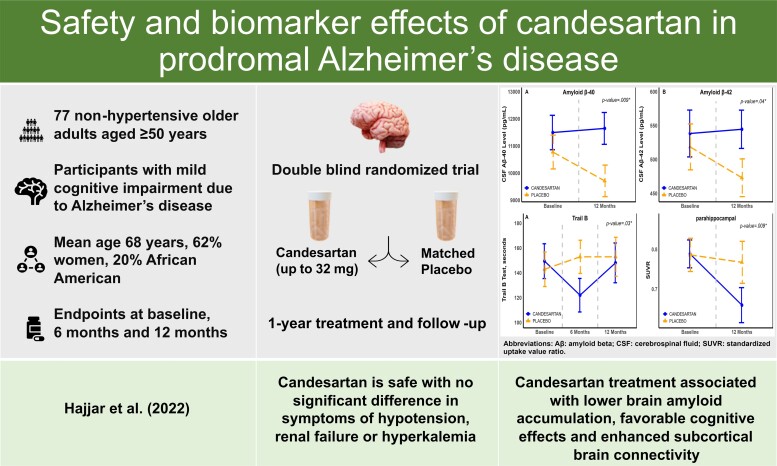

**See Yasar (https://doi.org/10.1093/braincomms/fcac293) for a scientific commentary on this article.**

## Introduction

The renin angiotensin system (RAS) plays a role in cognition and neurodegeneration.^1^ We have previously reported that candesartan, an angiotensin receptor blocker (ARB) that modulates RAS, has positive neurocognitive effects especially on executive function in those with hypertension and mild cognitive impairment (MCI).^2,3^ However, because of their effect on blood pressure and the lack of data on their cognitive effects in non-hypertensive individuals, it remained uncertain if these neurocognitive benefits are related to their blood pressure hemodynamic effects or a separate pleiotropic mechanism.

ARBs have a pleiotropic effect that is related to their selectively blocking angiotensin receptor type 1 (AT1) but not angiotensin receptor type 2 (AT2). AT1 activation results in vasoconstriction, endothelial dysfunction and smooth muscle hypertrophy.^4^ Whereas AT2 activation decreases superoxide production, activates neuronal repair systems by promoting neuronal cell differentiation and neurite growth, decreases inflammation and axonal degeneration, and may positively affect cognition.^5-8^ This effect, termed the AT2 hypothesis, can be leveraged to develop treatments for Alzheimer’s disease. In addition, ARBs may uniquely activate the ACE2/MAS and angiotensin IV axes, and both may exhibit neurocognitive protection.^9-11^ This remains to be shown, as the evidence for the neurocognitive effects of ARBs is derived from hypertensive populations as it has been predominantly used for managing hypertension.^12-14^

A key step to advance the potential for using ARBs in general and candesartan in particular for Alzheimer’s disease therapy is to demonstrate its safety and efficacy especially in engaging the amyloid cascade in non-hypertensive individuals. Previous preclinical animal and human autopsy studies have suggested that modulation with ARBs may affect Alzheimer’s disease biomarkers.^15-17^ Few prior studies have focused on candesartan and to the authors’ knowledge, no clinical trial has investigated the effects of candesartan on CSF biomarkers. Given the evolution of relying on CSF amyloid-β and tau biomarkers in enhancing diagnostic accuracy of the early stages of Alzheimer’s disease, we aimed to conduct this study in biomarker-positive Alzheimer’s disease individuals.

Hence, the objective of this double-blind randomized placebo-controlled trial was to assess the safety, tolerability, and effects of candesartan compared with placebo on Alzheimer’s disease biomarkers following 1-year treatment in non-hypertensive older adults with MCI due to Alzheimer’s disease (MCI-AD), also termed prodromal Alzheimer’s disease.

## Materials and methods

### Participants

Adult non-hypertensive participants aged 50 years or older with MCI and evidence of Alzheimer’s disease biomarker positivity (CSF amyloid-β42 (Aβ42), total tau or phosphorylated tau_181_ (p-tau_181_) levels fit the diagnostic criteria of prodromal Alzheimer’s disease based on published and validated Alzheimer’s disease neuroimaging initiative (ADNI) cutoffs^18^ or amyloid PET) were enrolled in the CEDAR (Candesartan's effects on Alzheimer’s disease and related biomarkers) trial. MCI was defined using the Petersen *et al*.^19^ criteria as the presence of: (i) subjective memory concern; (ii) Montreal Cognitive Assessment (MoCA) score <26; (iii) clinical dementia rating (CDR) global score and memory box score of 0.5; (iv) abnormal memory function documented using the education-adjusted Wechsler Memory Scale-IV Logical Memory Delayed Recall, paragraph A only; and (v) preservation of general functional abilities reflected by the functional activities questionnaire (FAQ) < 9. Supplementary Table 1 provides additional inclusion and exclusion data.

The CEDAR trial was approved by the Emory University Institutional Review Board (IRB number: IRB00084574). Written informed consent was obtained from all study participants prior to partaking in study activities in accordance with the principles of the Declaration of Helsinki. A study partner was mandatory to provide consent for participants who were lacking decision capacity. Investigational drugs were purchased through grant funding and supplied by the Emory University Investigational Drug Services. All study activities were overseen by an independent data and safety monitoring board (DSMB).

### Study design

**Figure 1:**
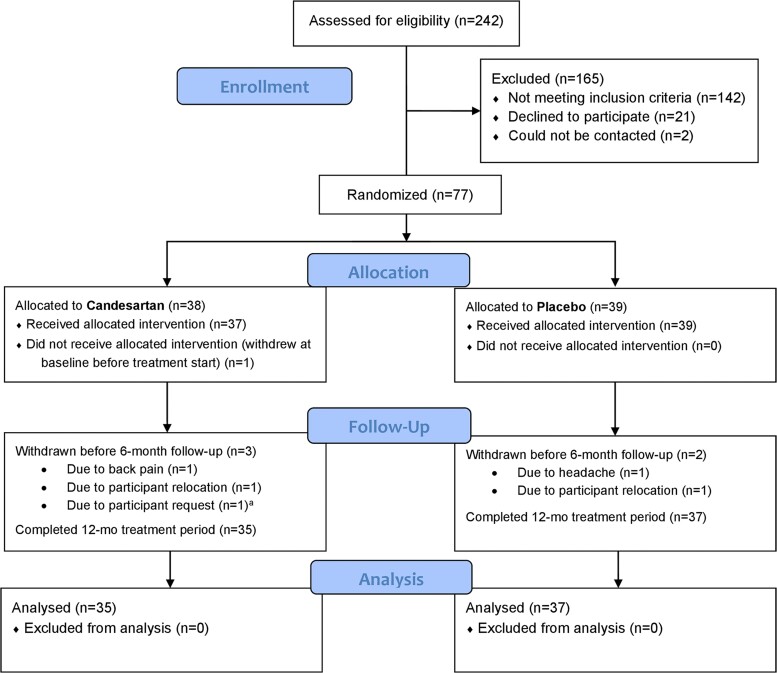
**Study enrollment, randomization and follow-up.** The consolidated standards of reporting trials (CONSORT) flow diagram for the CEDAR trial. ^a^Participant request to withdraw from study due to busy life schedule.

CEDAR was a 1-year, single-center, randomized, double-blind placebo-controlled trial comparing the effects of candesartan versus matched placebo in 77 eligible individuals in Metro Atlanta, who had prodromal Alzheimer’s disease and were neither diagnosed with nor were on treatment for hypertension. Following a screening visit, clinical assessment and lumbar puncture, enrolled participants were randomly allocated using a 1:1 ratio to either candesartan (intervention) or placebo (control) treatment groups for 12 months and stratified by use of cholinesterase inhibitors or memantine (taking versus not taking). All participants were commenced on 8 mg of oral candesartan or a matching placebo once daily, formulated into identical capsules, with as-needed dose escalations (candesartan 8 mg→16 mg→32 mg or matched placebo) every 2 weeks until maximal dose was achieved, or participant became symptomatic. In the latter case, the dose was de-escalated to a tolerable dose. Both groups underwent study medication dose escalations unless blood pressure readings were <100/40 mmHg or participant experienced hypotensive symptoms. Supplementary Table 2 shows the number of participants in each maximal tolerated dose group. Outcome measures were collected by trained study personnel at baseline, 6 months and 12 months. Study participants also had follow-up encounters during medication adjustment (titration) visits and at 3 and 9 months. Safety was monitored at all visits by standard laboratory tests, adverse event reporting and blood pressure measurements. Additional safety visits or phone assessments occurred as necessary. Study enrollment started on 30 June 2016, and all study participation was concluded by 17 August 2020. See CONSORT chart (Fig. 1) for study enrollment, drug allocation and study follow-up.

### Randomization and masking

An independent biostatistician and the Director of Emory Investigational Drug Services (IDS) provided oversight of randomization fidelity and blinding. To ensure equal distribution between the two groups on the important confounder of Alzheimer’s disease symptomatic therapy, we used block randomization via a computerized random number generator (SAS, V9.4), and stratified by use of cholinesterase inhibitor and memantine (taking versus not taking). Random allocation (1:1 ratio) of candesartan or the matching placebo was prepared by the Emory IDS pharmacy. See Supplementary Table 2 for study drug and dose allocation. Only the pharmacy and the unblinded statistician had access to randomization lists. Study participants, study investigators and research staff remained blinded to treatment allocation for the duration of study participation. From the initiation of the trial until the final completed analysis, all current authors and research personnel were blinded to the treatment group assignment. The unblinding of the results occurred after the last DSMB meeting.

### Procedures and outcomes

Study visit schedule and procedures are outlined in the trial protocol (Supplementary material). Safety assessments included medical history and physical examinations, including BP measurements (using automatic calibrated blood pressure machines with appropriate cuff size and placement),^20^ and laboratory testing for hyperkalemia and renal insufficiency. Lumbar punctures were performed at baseline and 12 months using 24G Sprotte atraumatic spinal needles and following a minimum of 6 h fast. To assess Alzheimer’s disease biomarker engagement, all samples were analysed for CSF Alzheimer’s disease biomarkers (Aβ42, Aβ40, total tau and p-tau_181_) on the Fujirebio Lumipulse platform using validated Fujirebio immunoassay reagents.^18^

The primary outcomes for our study were safety measures and Alzheimer’s disease biomarkers related to amyloid-β and tau brain pathology. Safety endpoints were defined as the number of hypotensive episodes (sitting BP <100/40 mmHg, measuring the mean of 2 readings taken 5 min apart), the number of participants with hypotensive symptoms (dizziness, weakness or fatigue, lightheadedness), the number of participants with elevated serum creatinine >2.5 mg/dl, the number of participants with elevated serum potassium >5.9 mEq/dl, the number of participants who reported adverse events, and the number of discontinuations of study medication due to any reason or deaths. Alzheimer’s disease biomarker outcomes included CSF measures of Aβ42, Aβ40, total tau and p-tau_181._

The study cognitive battery was collected at baseline, 6 months and 12 months. Executive functioning was assessed using the trail making test (TMT), Parts A and B.^21^ TMT Part B-A was also calculated, adjusting for motor speed and visuo-perception in measuring this domain. Participants unable to complete TMT Part B in 5 min were scored the maximum of 300 s. The NINDS-initiated computer-based EXecutive Abilities: Measures and Instruments for Neurobehavioral Evaluation and Research test yielding a single executive composite score further assessed executive functioning.^22^ Hopkins verbal learning test-revised (HVLT-R) is a 12-item list learning test used to assess episodic memory in older adults.^23^ HVLT-R measures collected include immediate and delayed recall. All participants and their assigned study informants were interviewed for a CDR scale and a sum of boxes (CDR-SB) assessing global cognition^24^ was derived for each participant at baseline and at 12 months. The digit span test (forward and backward) measured attention^25^ and the Boston Naming Test assessed language via the ability to name 15 visual confrontation drawings.^26^

In addition to the individual cognitive measures, we calculated a cognitive composite score from standardized *Z*-scores constructed from raw scores of 8 cognitive measures—TMT Part A and Part B, HVLT-R delayed recall, Boston Naming Test and Digit Span Test (forward and backward). This statistical approach serves to reduce potential type 1 error due to multiple testing, as well as examine a full range of abilities across cognitive domains among individuals at risk of developing Alzheimer’s disease.^27^ Functional capacity was assessed using the Instrumental activities of daily living (IADL) scale^28^, and depressive symptoms were evaluated using the Center for Epidemiologic Studies Depression scale.^29^

### Neuroimaging measures and analyses

In addition to CSF measures of Aβ and tau markers*, in vivo* amyloid and tau positron emission tomography (PET) imaging assessing deposition of Aβ and tau proteins in whole brain were conducted using ^11^C-PiB and ^18^F-flortaucipir radioligands, respectively.^30,31^ PET images were acquired for 20 min (4 × 5 min frames) approximately 50 min after intravenous administration of 14.81 +/−0.88 mCi of ^11^C-PiB or approximately 80 min after intravenous administration of 9.72 +/−0.69 mCi of ^18^F-flortaucipir. All participants were offered amyloid and/or tau PET scans, and images of participants who received both scans were collected on separate days. PET images were acquired at baseline and 12 months.

PET data were analysed using the following processing steps: images were smoothed with 10 mm full with at half max Gaussian kernel, resampled to 2 × 2 × 2 mm resolution and averaged across the frames using AFNI software package.^32^ Preprocessed data were further analysed using the PETSurfer processing pipeline (https://surfer.nmr.mgh.harvard.edu/fswiki/PetSurfer). Specifically, we co-registered PET and T1-weighted scans for each subject, and then used individual previously generated^33^ high-resolution segmentations (which included cortical and subcortical (SC) structures as well as extra cerebral regions) to perform partial volume correction (PVC) and computation of regional standardized uptake value ratios (SUVR). For PVC, we used the symmetric geometric transfer matrix with estimate of the point spread function of 8 mm full width at half maximum.^34^ Seven participants did not have a T1-weighted scan and therefore were excluded from the above analysis.

MRI was performed at baseline and 12 months on a 3T MRI scanner (Magnetom Prisma, Siemens, Erlangen, Germany) using a 20-channel head coil. The high-resolution T1-weighted image sequence was acquired using the magnetization-prepared rapid gradient-echo (MPRAGE), and resting state Functional MRI (rs-fMRI). rs-fMRI data analysis processing was performed in BrainForge^35^ via a containerized version of the GIFT software package (http://trendscenter.org/software/gift) in order to perform spatially constrained ICA analysis with 53 pre-defined component maps using the fully automated NeuroMark method.^36^ The resulting ICA components were written out for each subject using three measures: component spatial map, component power spectra, and between component connectivity (functional network connectivity, FNC). The FNC values were averaged across components comprising a given network (for example, thalamus, caudate, putamen and sub/hypothalamus components were averaged together for the SC network composite). These outcome variables were then tested within the MANCOVAN tool within GIFT for a difference between the treatment and the placebo groups.

### Statistical analysis

Baseline demographics and clinical characteristics of the two groups were compared for randomization fidelity using Chi-square test and Student *t*-test or Wilcoxon signed-rank test, and descriptive statistics presented as a count (%) or mean (SD). Chi-square test compared primary safety outcomes between the candesartan and placebo groups. The outcome analyses of estimated between-group differences in the change in study end points from baseline to 12 months were performed on data from this intention-to-treat population. We utilized mixed models with repeated measures (MMRM) with unstructured covariance to compare Alzheimer’s disease CSF biomarkers, neuroimaging and cognitive outcomes between the candesartan and placebo groups. The treatment effects on the study outcomes were derived from MMRM with a treatment group by visit (group*time) interaction term and adjusted for the stratification variable (use of cholinesterase inhibitors or memantine). Model-derived least square mean (LSM) and mean differences (MDs) by group and time with 95% confidence interval were derived from the MMRM. To account for multiple testing in the neuroimaging analyses, false discovery rate correction was performed using Benjamini-Hochberg procedure. Statistical analyses were performed using SAS 9.4 (Cary, NC, USA).

### Data availability

The study data, including deidentified participant data, can be made available to investigators upon request after publication of trial findings. Requests may be made by contacting the corresponding author. The study protocol and statistical analysis plan are available in the online Supplementary material.

## Results

### Participants

Of 242 persons screened, 165 were not eligible (142 did not meet cognitive, biomarker or blood pressure criteria, 23 declined or were not reachable after screening), and 77 were randomized to candesartan (*n* = 38) or placebo (*n* = 39). Of those randomized, five participants dropped out of the study before the 6-month visit (Fig. 1 and Supplementary Table 1). Demographics and clinical characteristics are presented in Table 1. Baseline features were comparable between both treatment groups, with no statistical differences in age, sex, years of education, cognitive status, blood biochemistry levels or co-morbid illnesses. Successful escalation into maximum dose (32 mg candesartan or matching placebo) was achieved in 58% of the enrolled sample (40% in candesartan and 77% in placebo). The distribution of doses per treatment group is provided in Supplementary Table 2.

**Table 1:** Demographic and clinical characteristics of participants at baseline^a^

	Candesartan (*N* = 38)	Placebo (*N* = 39)
**Demographics**		
Age, mean (SD), years	66.7 (8.4)	69.5 (8.5)
Female, *n* (%)	23 (60.5)	25 (64.1)
Ethnicity		
Non-Hispanic or Latino, *n*(%)	36 (94.7)	39 (100.0)
Hispanic or Latino, *n*(%)	2 (5.3)	0 (0.0)
Race		
White, *n* (%)	30 (78.9)	32 (82.1)
Black or African American, *n*(%)	8 (21.1)	7 (17.9)
Years of education, mean (SD)	15.9 (3.2)	15.3 (2.6)
Body mass index in Kg/m^2^, mean (SD)	25.3 (5.0)	26.7 (6.1)
**Blood pressure and heart rate, mean (SD)^b^**		
Sitting systolic BP, mmHg	124.7 (13.6)	126.7 (13.2)
Sitting diastolic BP, mmHg	67.9 (9.9)	69.8 (10.1)
Sitting heart rate, beats/min	66.3 (11.1)	67.3 (11.5)
Standing systolic BP, mmHg	128.5 (14.5)	130.9 (15.1)
Standing diastolic BP, mmHg	75.7 (9.4)	79.5 (10.8)
Standing heart rate, beats/min	73.6 (12.8)	75.1 (14.0)
**Cognitive status, mean (SD)**		
MoCA score	20.9 (3.9)	20.7 (2.7)
Logical memory, delayed	5.7 (4.9)	5.9 (5.0)
FAQ score	1.7 (1.9)	1.2 (1.7)
CDR, global score	0.5 (0.0)	0.5 (0.1)
CDR-SB	1.9 (0.9)	1.9 (1.0)
**Blood chemistry, mean (SD)**		
WBC (1000/ul)	5.9 (1.9)	5.7 (1.4)
Haemoglobin (g/dl)	13.6 (1.2)	14.0 (1.3)
Platelets (1000/ul)	242.4 (62.1)	237.3 (64.8)
Sodium (mmol/l)	140.6 (2.4)	140.3 (2.5)
Potassium (mEq/l)	4.5 (0.5)	4.5 (0.5)
Creatinine (mg/dl)	0.9 (0.2)	0.9 (0.2)
**Clinical diagnosis**		
Diabetes mellitus	1 (2.6)	2 (5.1)
Heart disease (coronary or valvular)	7 (18.4)	7 (18.0)
Hyperlipidemia	9 (23.7)	12 (30.8)
Depression^d^	9 (23.7)	11 (28.2)
**Pre-randomization medications**		
Cholinesterase inhibitors/Memantine	18 (47.4)	19 (48.7)
Cardiovascular drugs^e^	4 (10.5)	6 (15.4)
Antidiabetic drug	1 (2.6)	2 (5.1)
Lipid lowering agent	8 (21.1)	15 (38.5)
Antidepressant	15 (39.5)	19 (48.7)

BP, blood pressure; CDR-SB, clinical dementia rating—sum of boxes; FAQ, functional activities questionnaire; GED: General Educational Development; MoCA, Montreal Cognitive Assessment; SD, standard deviation; WBC, white blood cells. ^a^Data are reported as *n* (%) or mean (SD). ^b^Average sitting BP values are the mean of 2 sitting BP readings taken 5 min apart; standing BP values are BP readings taken after 3 min of standing. ^c^Remote stroke occurring >3 years from study enrollment. ^d^Clinically diagnosed depression. ^e^Used for indications other than hypertension.

### Safety

Overall, only five participants had symptomatic hypotensive episodes (four in candesartan versus one in placebo, *P* = 0.45). Although the number of participants who had at least one episode of BP ≤100/40 mmHg with or without symptoms was higher in the candesartan arm (16 versus 4, *P* = 0.001), the majority had no more than one or two episodes over the 1 year. One participant discontinued study medication and participation at 2 months due to adverse event of headache, and another participant had an elevated serum potassium >5.9 mEq/dl at 12 months which returned to normal levels following a repeat test. Both discontinued participants were in the placebo arm. None of the participants had impaired kidney function reflected by an elevated serum creatinine >2.5 mg/dl. These results are described in Table 2.

**Table 2:** Safety outcomes and adverse event summary

	Candesartan (*N* = 38) *n* (%)	Placebo (*N* = 39) *n* (%)	*P-value*
Outcome			
All hypotensive episodes^a^	16 (42.1)	4 (10.3)	0.001^b^
Hypotensive symptoms^c^	7 (18.4)	10 (25.6)	*0*.*45*
Hypotensive episodes and symptoms	4 (10.5)	1 (2.6)	*0*.*20*
Serum potassium > 5.9 mEq/dl	0 (0.0)	1 (2.6)	*0*.*99*
Serum creatinine > 2.5 mg/dl	0 (0.0)	0 (0.0)	*NA*
Discontinuation of study medication	0 (0.0)	1 (2.6)	*0*.*99*
Adverse events reported	23 (60.5)	22 (56.4)	*0*.*71*
Number of hypotensive episodes^a^			
1 hypotensive episode	7 (18.4)	2 (5.1)	0.99
2 hypotensive episodes	6 (15.8)	2 (5.1)	0.99
4 hypotensive episodes	1 (2.6)	0 (0.0)	0.99
5 hypotensive episodes	2 (5.3)	0 (0.0)	0.99

a Hypotensive episode was defined as sitting blood pressure (BP) < 100/40 mmHg, measuring the mean of 2 readings taken 5 min apart with or without symptoms during in person visits. ^b^Significance level derived from Chi-square test; all other *P*-values derived from Fisher’s exact test. ^c^Symptoms of hypotension included dizziness, weakness, fatigue and lightheadedness.

When considering sitting and 3 min standing blood pressure and heart rate readings and serum creatinine and potassium during the study period, we observed no differences between participants in the candesartan and placebo groups at baseline, 6 months, and 12 months (Supplementary Table 3 and Fig. 1). There were no differences in adverse events between both treatment groups during the study period (Supplementary Table 4). Twenty-three participants in the candesartan group reported at least one AE compared with 22 participants in the placebo group (60.5% versus 56.4%; *P* = 0.71). The most common reported adverse events for candesartan and placebo were dizziness (6 (16%) versus 5 (13%)), and fatigue, tiredness or weakness (4 (11%) versus 4 (10%)). No related serious adverse events or deaths were reported during this trial.

### CSF Alzheimer’s disease biomarkers

**Figure 2:**
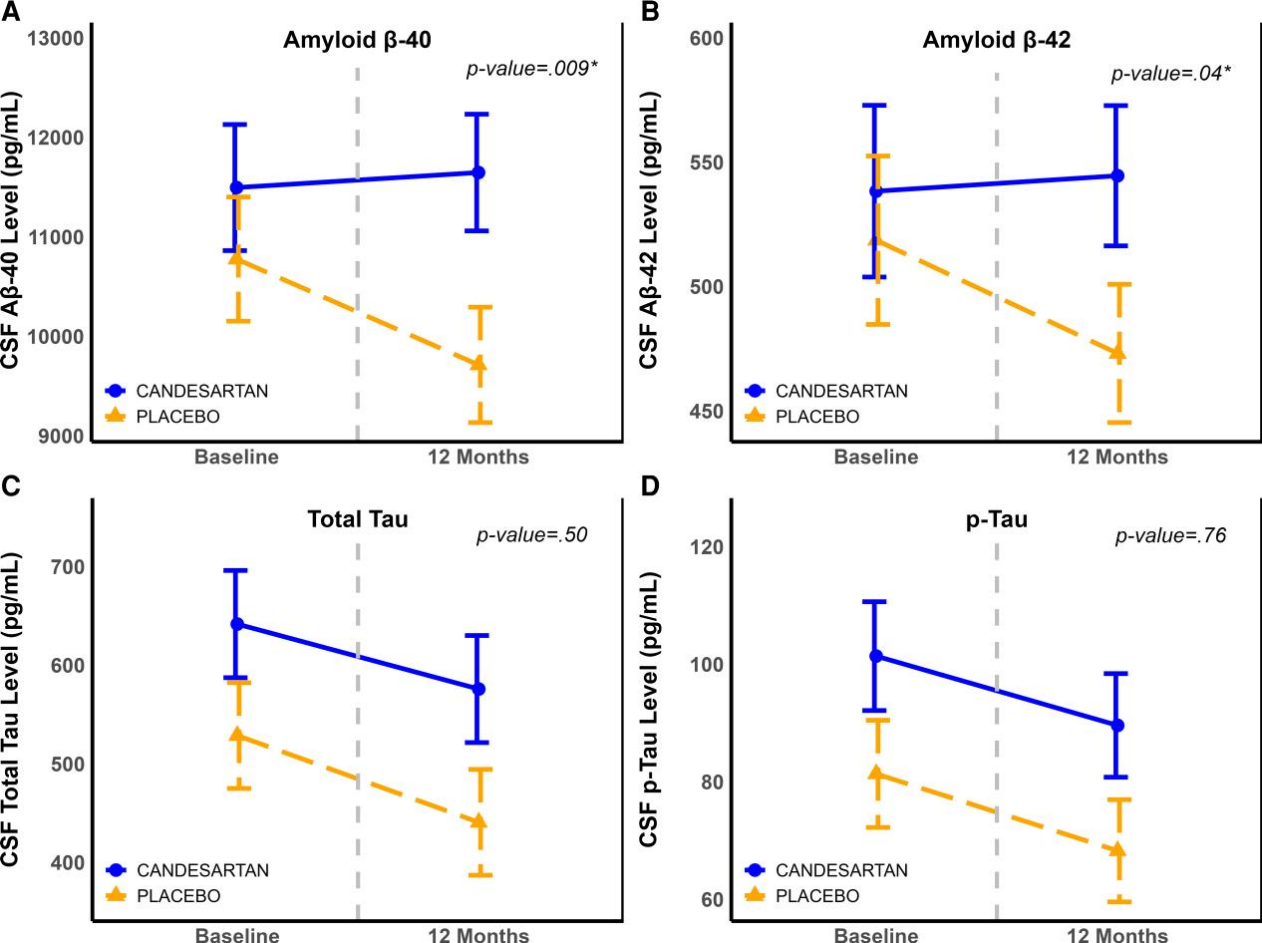
**Changes in cerebrospinal fluid Alzheimer’s disease Biomarkers over 12 months, by treatment group.** Panels (**A–D**) portraying changes in CSF Aβ40 (**A**), Aβ42 (**B**), total tau (**C**) and p-tau_181_ (**D**) levels measured at baseline and 12 months. These levels are illustrated by treatment group (Candesartan versus placebo). Values are model-derived least square means and standard errors (error bars), and are obtained from mixed model repeated measure for the interaction of treatment effect over time, and adjusted for use of cholinesterase inhibitors or memantine. See Supplementary Table 5 for details of treatment effect sizes and *P*-values.

Treatment with candesartan was associated with increases in CSF Aβ40 and Aβ42 levels reflecting lower brain amyloid accumulation whereas the placebo arm experienced further decreases in both measures: between-group MD Aβ40: 1211.95 pg/ml (95% CI: 313.27, 2110.63, *P* = 0.009) and MD of Aβ42: 49.51 pg/ml (95% CI: 98.05, 0.98, *P* = 0.046). A similar trend was observed with Aβ42/Aβ40 (MD: 0.001, 95% CI: −0.005, 0.007). There was no significant difference between the two groups in total tau or p-tau_181_. These results are provided in Fig. 2 and Supplementary Table 5.

### Neuroimaging results

**Figure 3:**
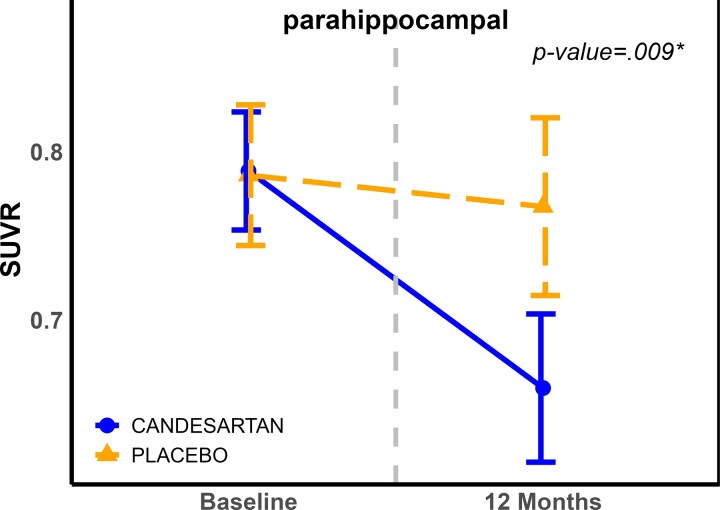
**Changes in SUVR in parahippocampal region over 12 months, by treatment group.** Values are model-derived least square means and standard errors (error bars) and are adjusted for use of cholinesterase inhibitors or memantine (*: FDR corrected). *P*-values for the treatment effect are derived from the mixed model repeated measure comparing change over the 1-year study period. Abbreviations: FDR, false discovery rate; SUVR, standardized uptake value ratio.

*PET scan results*: Although there was no significant effect of candesartan on global SUVr for both ^11^C-PiB and^18^ F-flortaucipir, regional analyses showed a decrease in ^11^C-PiB in the parahippocampal region [MD = −0.1104, 95% CI (−0.19, −0.029)]. See Fig. 3 and Supplementary Fig. 2. The raw *P*-value = 0.0085, which remained significant after FDR correction. No regional effects were observed on ^18^F-flortaucipir PET.

**Figure 4:**
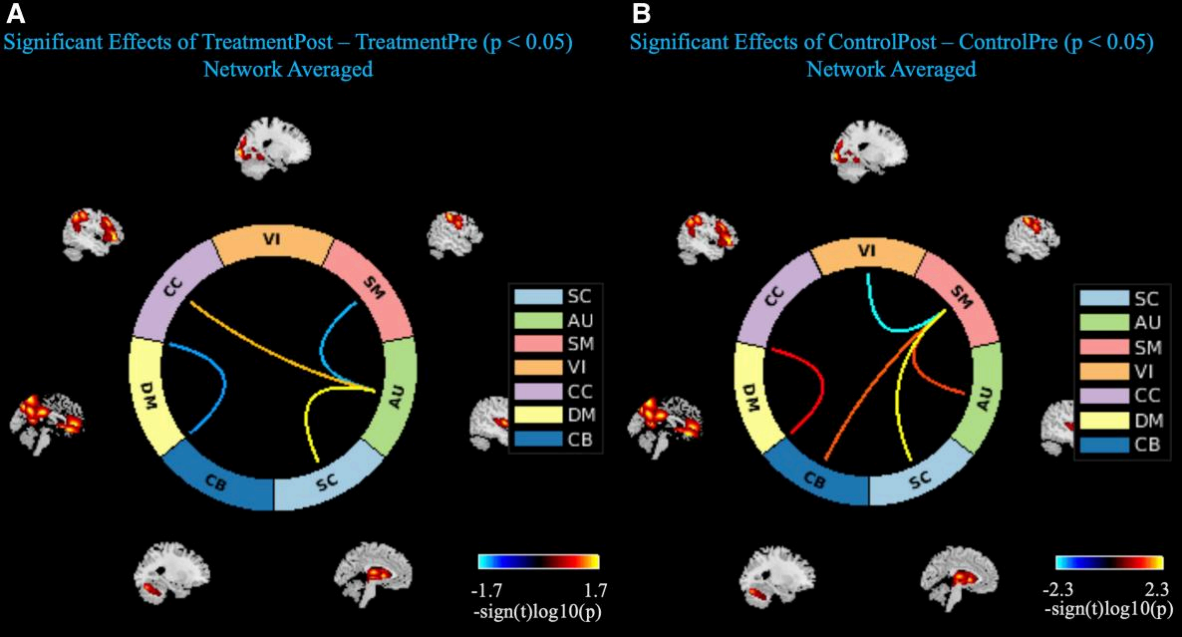
**Connectograms of significant changes in associated brain networks, by treatment group.** Candesartan group connectogram (**A**), Placebo group connectogram (**B**). Lines indicate associations between brain networks that were significantly changed from baseline to 12 months at *P*-value < 0.05. Network area abbreviations: AU, auditory; CB, cerebellar; CC, cognitive control; DM, default mode; SC, subcortical; SM, sensorimotor; VI, visual.

**Figure 5:**
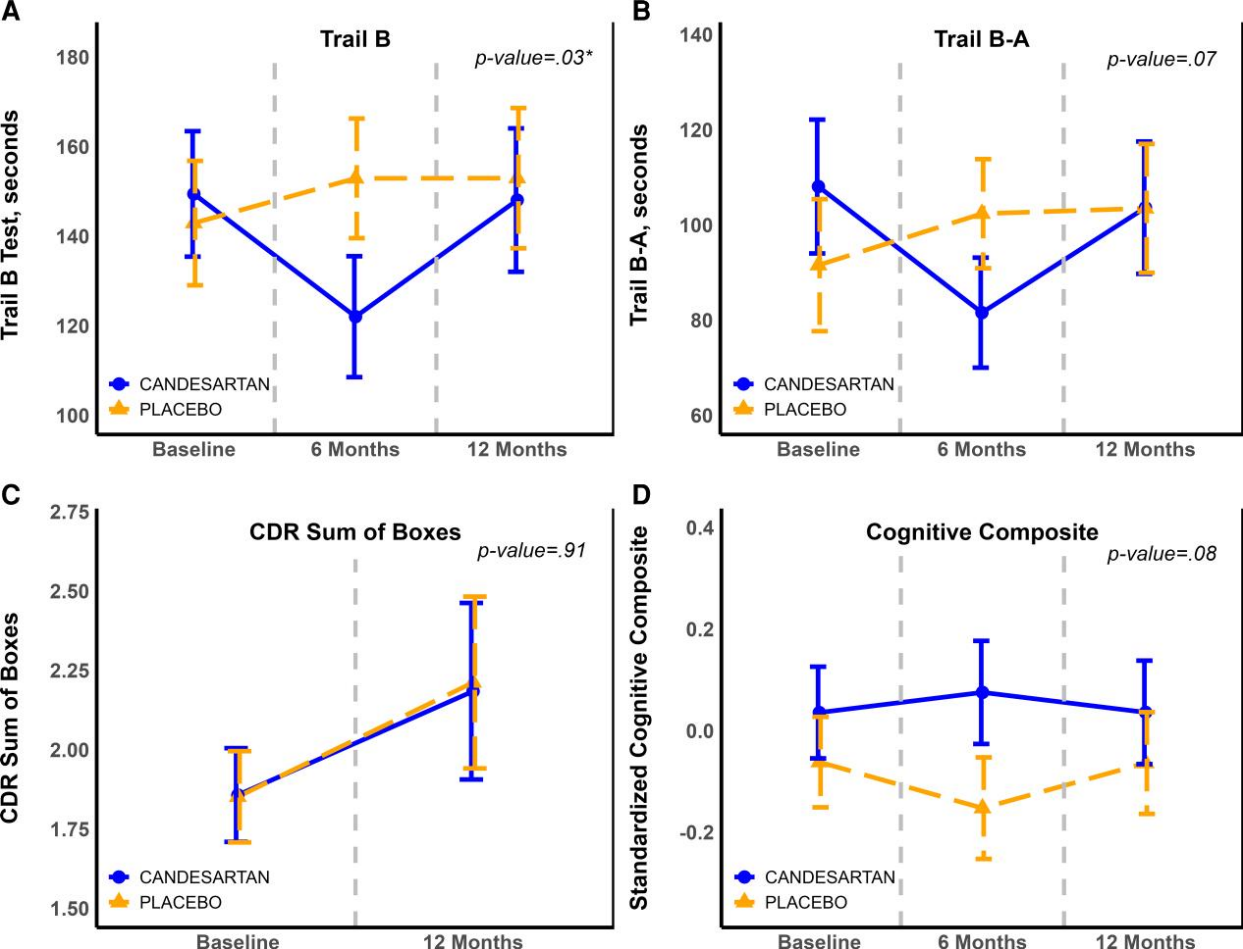
**Changes in exploratory cognitive outcomes over 12 months, by treatment group.** Panels (**A–D**) portraying changes in exploratory cognitive outcome measures, i.e. Trail Making Test (TMT) Part B (**A**), TMT Part B-A (**B**), CDR Sum of Boxes score (**C**), and the standardized cognitive composite (**D**) measured at baseline, 6 months and 12 months. These levels are illustrated by treatment group (Candesartan versus placebo). Values are model-derived least square means and standard errors (error bars), and are obtained from mixed model repeated measure for the treatment effect over time, and adjusted for use of cholinesterase inhibitors or memantine. See Supplementary Table 6 for details of treatment effect sizes and *P*-values. Abbreviations: CDR, Clinical dementia rating; TMT, Trail making test.

*MRI results*: Treatment with candesartan was associated with differential effects on brain network connectivity. Candesartan was associated with increased domain-averaged FNC of the SC with auditory (AU) network areas (*t*-stat = 2.465, *P* = 0.019), as well as the cognitive control (CC) with AU network connectivity (*t*-stat = 2.234, *P* = 0.033). In the candesartan group, there was also a simultaneous decrease in FNC with the AU and sensorimotor (SM) network areas (*t*-stat = −2.168, *P* = 0.038) (Fig. 4A). This trend is contrasted with the placebo group where the AU and SM averaged network connectivity increased (*t*-stat = 2.404, *P* = 0.022), and the default mode network averaged network interconnectivity increased from baseline to 12 months (*t*-stat = 2.199, *P* = 0.035) (Fig. 4B). There was no significant effect of treatment on hippocampal volume between both treatment groups. These results are shown in Supplementary Table 6.

### Cognitive function results

Treatment with candesartan was associated with a statistically significant improvement of TMT Part B performance compared with a conversely worsening performance in the placebo group by 12 months (MD: −11.41 s, 95% CI: −11.94, −10.89, *P* = 0.03). A similar trend was observed for TMT Part B-A (−16.36, 95% CI: −17.13, −15.6, *P* = 0.07). Compared with placebo, there was an overall trend for better cognitive performance in the candesartan group reflected by the composite score (MD: 0.002, 95% CI: −0.0002, 0.005, *P* = 0.076). These results are shown in Fig. 5. There were no significant differences observed between the two treatment groups for the other cognitive outcomes measured during the trial period, including CDR-SB and IADL. These results are shown in Supplementary Table 6.

## Discussion

In this study of non-hypertensive individuals with prodromal Alzheimer’s disease, candesartan was safe and associated with decreased amyloid markers in CSF and in the hippocampal region on amyloid PET imaging, improved executive function and enhanced brain connectivity in multiple brain networks. In addition, the use of candesartan was associated with a trend towards an improved derived score of global cognition.

Animal and human observational studies have explored the associations between neuropathological expressions of Alzheimer’s disease and ARB drugs such as losartan, valsartan, telmisartan, candesartan and olmesartan. RAS has been implicated in Alzheimer’s disease-related pathological mechanisms including the amyloid cascade in animal models. Previous research evidence has determined that the use of ARBs in general is associated with preserved cognitive function and lower postmortem markers of amyloid in autopsy series.^37^ However, not many studies have tested the effects of specific ARBs on Alzheimer’s disease pathology. Even fewer clinical trials have compared the unique pleiotropic properties of these in-class-sartan drugs. Although similar in their mechanistic effects on blood pressure control, studies have hypothesized that varying differences exist in their influence on neurodegenerative and cognitive outcomes.^38^ Since ARBs are almost exclusively used for managing hypertension, most prior evidence and our trials on candesartan have included only hypertensive individuals.^2,39-42^ This study is a first step towards repurposing candesartan for Alzheimer’s disease treatment independent of a hypertension status. Further, we have found that the use of candesartan in prodromal Alzheimer’s disease may not be associated with severe or clinically significant hypotension nor worsening cognitive function from lowering blood pressure in non-hypertensive individuals. There was also no evidence of worsening kidney function or hyperkalemia, the most clinically significant adverse events from using RAS modulating medications.

Few, if any, oral medications have been reported to lower brain amyloid levels. One recent observational study has suggested that use of ARBs is associated with lower Aβ accumulation over time in hypertensive individuals.^43^ This study provides evidence in a clinical trial setting that candesartan also has a favourable effect on amyloid biomarkers in Alzheimer’s disease. The underlying mechanisms remain to be explored. Although not tested specifically in this study, ARBs in general may impact many pathological mechanisms in Alzheimer’s disease including the neurovascular unit, neuroinflammation, neuronal cell survival, blood brain barrier integrity, endothelial dysfunction, and hemodynamic compromise.^44,45^ Candesartan may have unique effects in the brain that explain these trial results such as its effect on PPAR-gamma, ischemia/reperfusion protection, angiogenesis, extracellular matrix regulation and chromosomal maintenance.^46-49^

This study also confirms our prior findings of positive effects on executive function detected in hypertensive individuals. Although we did not see a specific effect on memory, overall cognitive functioning trended for a positive effect. It is possible to hypothesize that with a larger and longer study, the effects on memory may be more evident. This is supported by the findings from the Study on Cognition and Prognosis in the Elderly clinical trial in which candesartan significantly protected against declines in attention and episodic memory compared with placebo.^39^ However, this remains to be further confirmed in future studies.

Our prior observational study which analysed the longitudinal ADNI data showed a reduction in CSF total tau and p-tau_181_ among individuals taking ARBs.^50^ However, this study found no significant drug effects with tau measures in CSF or PET. A possible explanation may be that few, if any of the ADNI participants were on candesartan and our observed effect in this trial may be unique to this compound. We did not see any effect of candesartan on brain volumes. A recent study of losartan also failed to show an impact on measures of brain atrophy in Alzheimer’s disease dementia.^51^ Although our study duration was for 1 year—a period which may not be long enough to detect structural brain changes, it is possible that ARBs affect the earlier molecular changes in Alzheimer’s disease such as amyloid dysregulation rather than latter mechanisms such as tau phosphorylation and brain or hippocampal atrophy.

This CEDAR trial provides novel findings specific to candesartan in non-hypertensives whereas previous observational and clinical trial data compared other ARBs or were mostly focused on hypertensive individuals. This study provides the first in-human evidence that candesartan may possess Alzheimer’s disease-modifying characteristics in normotensive individuals which are likely independent of its blood pressure effects. A key potential criticism for our study is the small sample size. An initial step in repurposing a drug used in hypertension for Alzheimer’s disease is to demonstrate its safety on a small scale. The key factor that was considered when designing this study is the potential for harm from lowering blood pressure in prodromal Alzheimer’s disease. Prior observational studies have suggested that excessive lowering of blood pressure in older adults may be associated with worsening cognition.^52^ Hence, we intended to approach this at small-scale and provide critical evidence that when used in non-hypertensive individuals, there was no evidence of disease progression with candesartan. Despite its relatively small sample size, we were able to detect favourable effects on multiple Alzheimer’s disease indicators. This, along with our prior studies such as the CALIBREX trial (*n* = 177)^2^ provides further support to advancing candesartan in Alzheimer’s disease and related dementia therapeutics.

## Conclusion

In this study of prodromal Alzheimer’s disease, candesartan was associated with lower brain Aβ indicators and favourable neurocognitive and brain connectivity measures without any significant safety concerns. When combined with preclinical and clinical data from prior studies, these findings demonstrate that candesartan has a high potential for offering both disease-modifying and symptomatic effects in early Alzheimer’s disease. A larger trial to further validate these results is critical to repurpose candesartan as a therapeutic modality for Alzheimer’s disease.

## Supplementary Material

fcac270_Supplementary_Data

## Data Availability

Clinical trial deidentified data are available upon request from I.H.

## Abbreviations

Aβ =: amyloid beta
ACE2/MAS =: angiotensin converting enzyme 2/Mas receptor axis
AD =: Alzheimer’s disease
ARB =: angiotensin receptor blocker
AT2 =: angiotensin receptor type 2
CDR =: clinical dementia rating
C-PiB =: Pittsburgh compound-B
FDR =: false discovery rate
FNC =: functional network connectivity
HVLT-R =: Hopkins verbal learning test-revised
IADL =: instrumental activities of daily living
MCI =: mild cognitive impairment
MMRM =: mixed models with repeated measures
MoCA =: Montreal Cognitive Assessment
PPAR =: peroxisome proliferator-activated receptors
P-tau =: phosphorylated tau
RAS =: renin angiotensin system
SUVR =: standardized uptake value ratio
TMT =: trail making test
