# Supplementary material for: Safety and biomarker effects of candesartan in non-hypertensive adults with prodromal Alzheimer’s disease
Source: Brain Commun. 2022 Oct 25;4(6):fcac270. doi: 10.1093/braincomms/fcac270 (PMC9683395; doi:10.1093/braincomms/fcac270)
Supplement: fcac270_Supplementary_Data [file fcac270_supplementary_data.zip › Supplementary _material.docx]

**ONLINE SUPPLEMENTARY MATERIAL**

This supplement contains the following items:

- **Supplementary Table 1.** Reasons for CEDAR Study Ineligibility
- **Supplementary Table 2.** CEDAR Study Drug Allocation during 12-month Study Participation
- **Supplementary Table 3.** Blood Pressure, Pulse Rate and Blood Biochemistry during Study Period
- **Supplementary Table 4.** Adverse Events Reporting during CEDAR Study Period
- **Supplementary Table 5.** Treatment Effects of Cerebrospinal Fluid AD Biomarkers
- **Supplementary Table 6.** Treatment Effects of Cognitive and Structural Brain Measures
- **Supplementary Figure 1.** Serum Potassium and Creatinine during Study Period, by Treatment Group
- **Supplementary Figure 2.** Voxelwise Mapping of ^11^C-PiB Neuroimaging Greater in the Placebo compared to Candesartan
- **CEDAR clinical trial final protocol (version 14.0; March 23, 2020)**
- **Statistical analysis plan**

**Supplementary Table 1.** Reasons for CEDAR Study Ineligibility (n=165)

| **Study Ineligibility Reason** | **Total**  **(n=165)**  **No. (%)** |
| --- | --- |
| Cognitive status | 91 (55.2) |
| Lab/LP/PET results | 35 (21.2) |
| Declined study procedures | 21 (12.7) |
| Blood pressure related issues | 10 (6.1) |
| Medical reasons | 6 (3.6) |
| Loss to follow-up after screening | 2 (1.2) |

Abbreviations: LP: lumbar puncture; PET: positron emission tomography

**Supplementary Table 2.** CEDAR Study Drug Allocation during 12-month Study Participation

| **Study Drug Step and Dose^a^** | **Overall (N=77)**  **No. (%)** | **Candesartan (n=38)**  **No. (%)** | **Placebo  (n=39)**  **No. (%)** |
| --- | --- | --- | --- |
| Candesartan/Placebo 8 mg^b^ | 19 (24.7) | 15 (39.5) | 4 (10.3) |
| Candesartan/Placebo 16 mg | 13 (16.9) | 8 (21.0) | 5 (12.8) |
| Candesartan/Placebo 32 mg | 45 (58.4) | 15 (39.5) | 30 (76.9) |

^a^Between the 3 mo and 9 mo follow-up visits, 5 subjects’ study doses were stepped down. Of the 5, 3 were on candesartan, 2 were on placebo.

^b^4 subjects were on 8 mg every other day dosage

**Supplementary Table 3.** Blood Pressure, Pulse Rate and Blood Biochemistry Measures during CEDAR Study Period

| **Outcome^a,b^** | **Visit** | **Candesartan**  **(N=38)^c^**  **LSMean [95% CI]** | **Placebo**  **(N=39)^d^**  **LSMean [95% CI]** | ***Group*Time***  ***p-value*** |
| --- | --- | --- | --- | --- |
| Sitting systolic BP (mmHg), mean | Baseline | 124.0 [119.6, 128.4] | 126.7 [122.3, 131.0] | *.14* |
|  | 6 Month | 113.4 [107.5, 119.3] | 122.4 [116.6, 128.2] |  |
|  | 12 Month | 115.8 [110.65, 120.97] | 124.9 [119.81, 129.89] |  |
| Sitting diastolic BP (mmHg), mean | Baseline | 67.5 [64.31, 70.71] | 69.8 [66.67, 72.99] | *.59* |
|  | 6 Month | 63.6 [59.76, 67.5] | 67.7 [63.96, 71.52] |  |
|  | 12 Month | 65.3 [61.75, 68.89] | 69.4 [65.86, 72.85] |  |
| Sitting pulse (beats/min), mean | Baseline | 65.9 [62.5, 69.39] | 67.5 [64.08, 70.88] | *.18* |
|  | 6 Month | 67.9 [63.3, 72.5] | 69.9 [65.41, 74.42] |  |
|  | 12 Month | 68.3 [64.8, 71.85] | 66.9 [63.47, 70.37] |  |
| Standing systolic BP (mmHg), mean | Baseline | 127.7 [122.88, 132.59] | 130.7 [125.92, 135.51] | *.05* |
|  | 6 Month | 119.1 [112.98, 125.2] | 127.5 [121.5, 133.44] |  |
|  | 12 Month | 117.6 [112.6, 122.57] | 128.9 [124.02, 133.76] |  |
| Standing diastolic BP (mmHg), mean | Baseline | 75.5 [72.16, 78.75] | 79.3 [76.05, 82.55] | *.74* |
|  | 6 Month | 72.8 [68.56, 77.04] | 78.3 [74.13, 82.41] |  |
|  | 12 Month | 71.5 [68.15, 74.94] | 76.7 [73.38, 80.03] |  |
| Standing pulse (beats/min), mean | Baseline | 73.4 [69.28, 77.48] | 75.2 [71.1, 79.19] | *.13* |
|  | 6 Month | 73.2 [68.03, 78.3] | 77.8 [72.74, 82.81] |  |
|  | 12 Month | 75.3 [71.05, 79.53] | 74.3 [70.15, 78.45] |  |
| Serum potassium (mmol/L) | Baseline | 4.35 [4.21, 4.49] | 4.41 [4.27, 4.55] | *.67* |
|  | 6 Month | 4.55 [4.36, 4.73] | 4.56 [4.39, 4.74] |  |
|  | 12 Month | 4.58 [4.43, 4.74] | 4.53 [4.38, 4.69] |  |
| Serum creatinine (mg/dL) | Baseline | 0.88 [0.83, 0.94] | 0.9 [0.84, 0.95] | *.06* |
|  | 6 Month | 0.93 [0.86, 1.00] | 0.93 [0.87, 1.00] |  |
|  | 12 Month | 0.97 [0.9, 1.04] | 0.91 [0.84, 0.98] |  |

Abbreviations: BP: blood pressure; SD: standard deviation.

Values are lease square means derived from mixed models with repeated measures.

^a^Sitting systolic and diastolic BP and pulse rates are averages of 2 readings 5 min apart.

^b^Standing BP is after standing for 3 mins.

^c^Due to study drop-out, participants who completed 6-month study visits were 35 for candesartan and 37 for placebo.

^d^Due to study drop-out, number of participants who completed 12-month study visits were 35 for candesartan and 37 for placebo.

**Supplementary Table 4.** Related Adverse Events Reporting during CEDAR Study Period

| **Symptom** | **Candesartan**  **(n=38)**  **n (%)** | **Placebo**  **(n=39)**  **n (%)** |
| --- | --- | --- |
| Dizziness | 6 (16) | 5 (13) |
| Fatigue, tiredness, weakness | 4 (11) | 4 (10) |
| Headache | 2 (5) | 5 (13) |
| Back pain | 2 (5) | 0 (0) |
| Lightheadedness | 1 (3) | 1 (3) |
| Rash | 1 (3) | 0 (0) |
| Fall | 1 (3) | 0 (0) |
| Pain (back of head) | 1 (3) | 0 (0) |
| Panic attack | 1 (3) | 0 (0) |
| Vasovagal syncope | 1 (3) | 0 (0) |
| Joint pain | 0 (0) | 1 (3) |
| Runny nose | 0 (0) | 1 (3) |

**Supplementary Table 5.** Treatment Effects of Cerebrospinal Fluid AD Biomarkers^a,b^

| **Outcome** | **Visit** | **Candesartan**  **(n=36)**  **LSMean (SE)** | **Placebo**  **(n=37)**  **LSMean (SE)** | **Mean Difference**  **(95% CI)** |
| --- | --- | --- | --- | --- |
| Aβ40, pg/ml | Baseline | 11624.00 (625.86) | 10802.00 (623.82) | 1211.95  (313.27, 2110.63) |
|  | 12 Month | 11769.00 (571.54) | 9735.00 (573.74) |  |
| Aβ42, pg/ml | Baseline | 554.80 (34.09) | 523.03 (33.98) | 49.51  (98.05, 0.98) |
|  | 12 Month | 557.60 (27.32) | 476.32 (27.43) |  |
| Aβ42/Aβ40 | Baseline | 0.049 (0.003) | 0.052 (0.003) | 0.001  (-0.005, 0.007) |
|  | 12 Month | 0.050 (0.003) | 0.051 (0.003) |  |
| Total tau, pg/ml | Baseline | 638.31 (53.61) | 529.04 (53.50) | 21.44  (-42.21, 85.08) |
|  | 12 Month | 571.96 (52.89) | 441.25 (53.02) |  |
| Phospho-tau_181_, pg/ml | Baseline | 100.29 (9.09) | 81.58 (9.07) | 1.32  (-7.40, 10.04) |
|  | 12 Month | 88.52 (8.58) | 68.49 (8.59) |  |

Abbreviations: AD: Alzheimer’s disease; CSF: cerebrospinal fluid; Aβ: amyloid beta; LS Mean: Least Square Means; phospho-tau_181_: phosphorylated tau_181_; SE: standard error.

^a^Treatment effect size is derived from MMRM and adjusted for the use of cholinesterase inhibitors or memantine.

^b^Of total sample, 4 participants (2 in candesartan group, 2 in placebo group) did not have CSF data.

**Supplementary Table 6.** Treatment Effects of Cognitive and Structural Brain Measures^a^

| **Outcome** | **Visit** | **Candesartan**  **(n=38)**  **LS Mean (SE), n** | **Placebo**  **(n=39)**  **LS Mean (SE), n** | **Mean Difference [95% CI]**  ***p-value*** |
| --- | --- | --- | --- | --- |
| TMT Part A (seconds) | Baseline | 41.29 (3.64), 38 | 44.83 (3.59), 39 | -3.17 [-15.79, 9.45]  *.33* |
|  | 6 Month | 38.91 (4.26), 35 | 49.47 (4.21), 35 |  |
|  | 12 Month | 43.24 (5.52), 35 | 49.96 (5.39), 37 |  |
| EXAMINER executive composite | Baseline | 0.03 (0.11), 38 | -0.05 (0.11), 39 | 0.06 [-0.15,0.27]  *.76* |
|  | 6 Month | 0.18 (0.14), 35 | 0.04 (0.14), 36 |  |
|  | 12 Month | 0.07 (0.14), 35 | -0.06 (0.14), 37 |  |
| HVLT-R, delayed recall | Baseline | 4.83 (0.53), 37 | 5.29 (0.52), 39 | 0.18 [-0.72, 1.07]  *.38* |
|  | 6 Month | 5.40 (0.52), 35 | 5.08 (0.51), 36 |  |
|  | 12 Month | 5.10 (0.52), 35 | 5.39 (0.51), 37 |  |
| Digital Span Test (Backward) | Baseline | 5.72 (0.47), 38 | 5.35 (0.46), 39 | -0.38 [-1.57,0.82]  *.62* |
|  | 6 Month | 5.83 (0.46), 35 | 5.33 (0.46), 36 |  |
|  | 12 Month | 5.13 (0.44), 35 | 5.13 (0.43), 37 |  |
| Digital Span Test (Forward) | Baseline | 9.36 (0.37), 38 | 8.51 (0.37), 39 | 0.17 [-0.75,1.09]  *.08* |
|  | 6 Month | 9.10 (0.37), 35 | 9.04 (0.37), 36 |  |
|  | 12 Month | 9.94 (0.41), 35 | 8.92 (0.41), 37 |  |
| Boston Naming Test | Baseline | 13.73 (0.28), 38 | 13.31 (0.28), 39 | -0.13 [-0.86, 0.59]  *.48* |
|  | 6 Month | 13.71 (0.31), 35 | 13.12 (0.31), 35 |  |
|  | 12 Month | 13.47 (0.34), 35 | 13.18 (0.33), 37 |  |
| IADL | Baseline | 7.39 (0.17), 38 | 7.51 (0.17), 39 | -0.45 [-0.97, 0.06]  *.09* |
|  | 12 Month | 7.25 (0.25), 35 | 6.92 (0.24), 37 |  |
| Hippocampal volume, mm^3^ | Baseline | 6949.82 (151.66), 36 | 6662.21 (151.49), 34 | 47.79 [-83.02, 178.6]  *.47* |
|  | 12 Month | 6761.12 (151.42), 34 | 6521.30 (152.14), 31 |  |
| ^11^C-PiB, global SUVR | Baseline | 1.32 [1.23, 1.4], 26 | 1.42 [1.32, 1.52],20 | 0.02 [-0.08, 0.12]  *.71* |
|  | 12 Month | 1.34 [1.23, 1.45],24 | 1.46 [1.34, 1.59],18 |  |
| ^18^F-Flortaucipir, global SUVR | Baseline | 1.33 (0.05), 18 | 1.36 (0.04), 20 | -0.03 [-0.13, 0.07]  *.53* |
|  | 12 Month | 1.34 (0.06), 14 | 1.34 (0.05), 17 |  |

Abbreviations: EXAMINER: Executive Abilities: Methods and Instruments for Neurobehavioral Evaluation and Research; HVLT-R: Hopkins Verbal Learning Test - Revised; IADL: Instrumental Activities of Daily Living; LS Mean: Least Square Means; PiB: Pittsburgh compound B; SE: standard error; SUVR: standardized uptake value ratio; TMT: Trail Making Test.

^a^Least square means (standard error) and treatment mean differences were derived from mixed models with repeated measures (MMRM) and adjusted for use of cholinesterase inhibitors or memantine.

**Supplementary Figure 1.** Serum Potassium and Creatinine during Study Period, by Treatment Group


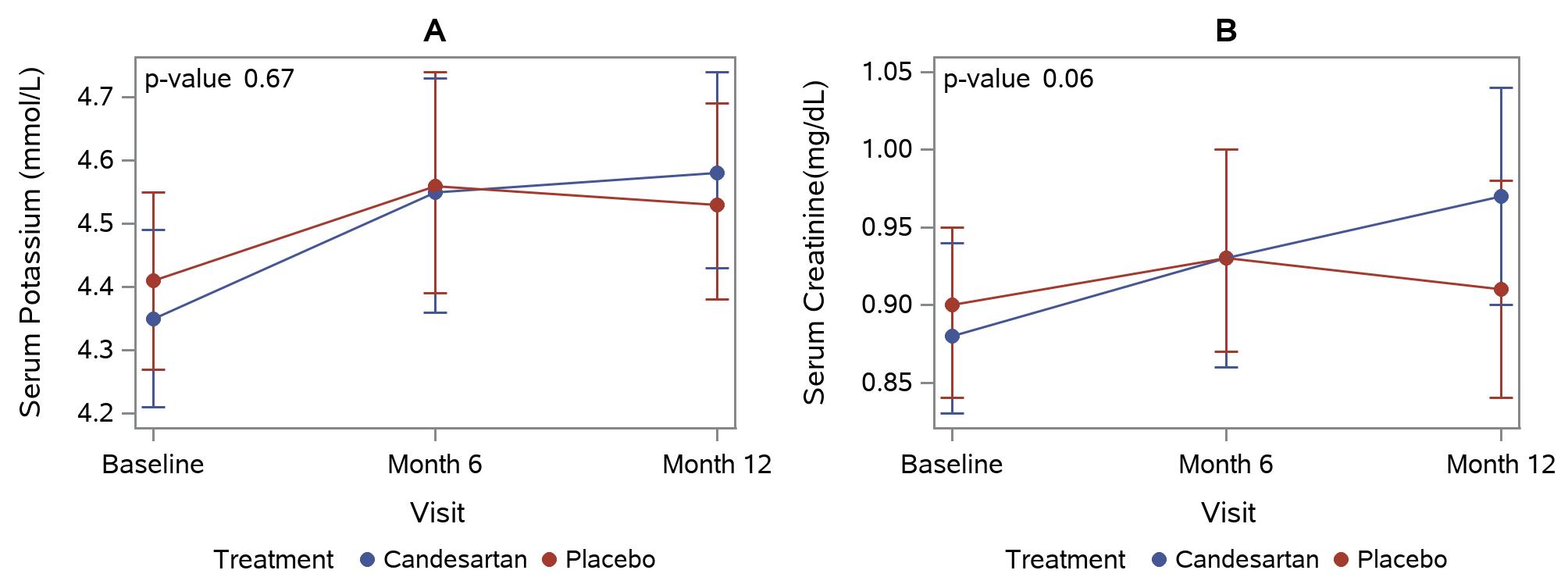


Serum potassium (panel A) and creatinine (panel B) measurements and standard errors (error bars) measured at baseline, 6 months, and 12 months are illustrated by treatment group. P-value is indicated for treatment effect by group (candesartan vs. placebo).

**Supplementary Figure 2.** Voxelwise Mapping of ^11^C-PiB Neuroimaging Greater in the Placebo compared to Candesartan


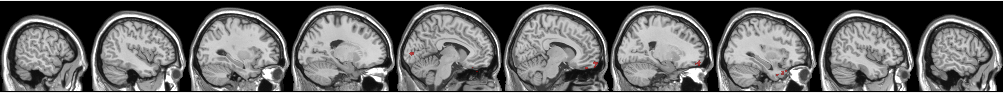

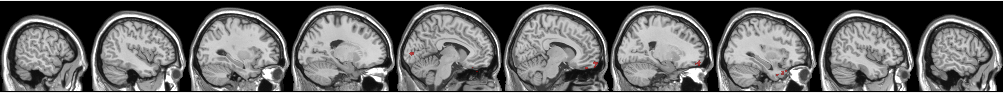


Voxelwise mapping shows brain regions (red) in which there is significantly greater ^11^C-PiB uptake in the placebo group compared to the candesartan group. Statistical t-maps are of significant voxel clusters greater than the critical t-value (t-critical=2.72) displayed on the MNI single subject brain.
