# Supplementary material for: Safety and biomarker effects of candesartan in non-hypertensive adults with prodromal Alzheimer’s disease
Source: Brain Commun. 2022 Oct 25;4(6):fcac270. doi: 10.1093/braincomms/fcac270 (PMC9683395; doi:10.1093/braincomms/fcac270)
Supplement: fcac270_Supplementary_Data [file fcac270_supplementary_data.zip › Supplementary_material_CEDAR_protocol.pdf]

Candesartan's Effects on Alzheimer's Disease And Related Biomarkers (CEDAR)

(ClinicalTrials.gov Identifier: NCT02646982)

This supplement contains the following items:

1. Final protocol (version 14.0; March 23, 2020)
2. Statistical analysis plan

PROTOCOL

Candesartan's Effects on Alzheimer's Disease And Related Biomarkers

(CEDAR)

VERSION 14; 3/23/2020

Principal Investigator:

Ihab Hajjar, MD

Emory University

TABLE OF CONTENTS

STUDY TEAM ROSTER 1

PARTICIPATING STUDY SITES 3

PRÉCIS 4

SCHEDULE OF PROCEDURES FOR THE DURTAION OF THE STUDY 5

1. STUDY OBJECTIVES 7

2. BACKGROUND AND RATIONALE 7

2.1. DisEASE targets for ARBs in AD 7
2.2. Safety of ARBs in non-hypertensive individuals 7
2.3. Study Rationale 8

3. STUDY DESIGN 8

4. SELECTION AND ENROLLMENT OF PARTICIPANTS 8

4.1. Inclusion Criteria 8
4.2. Exclusion Criteria 9
4.3. Study Recruitment 9

5. STUDY INTERVENTIONS 10

5.1. Interventions, Administration, and Duration 10
5.2. Handling of Study Interventions 10
5.3. Concomitant Intervention 10
5.3.1. Allowed Interventions *10*
5.3.2. Prohibited Interventions *10*
5.4. Adherence Assessment 10

6. STUDY PROCEDURES AND MEASURES 10

6.1. Questionnaires, lifestyle and anthropometric measures 11
6.2. Blood pressure measurement 11
6.3. Neuropsychological assessment: 11
6.4. Cerebrospinal Fluid (CSF) Collection 12
6.5. Brain MRI and PET imaging protocols 12
6.6. Vascular measures 13
6.7. Proteomics and inflammatory markers 13
6.8. Renin angiotensin system Activity 13
6.9. Blood chemistries, blood count, and APO-e 13
6.10. Blood will be drawn at various visits. Please refer to the Table for specific tests done at each visit. Blood and CSF Banking 13
6.11. Schedule of Evlauations 14
6.11.1. Screening *14*
6.11.2. Enrollment, baseline, and follow-up *14*

7. SAFETY ASSESSMENTS 14

7.1. Specification of Safety Parameters 14
7.2. Methods for assessing and recording Safety Parameters 15
7.3. Reporting Adverse Events and DEATH 15
7.4. Follow-up for Adverse Events 16
7.5. Incidental findings 16

8. INTERVENTION DISCONTINUATION 16

9. STATISTICAL CONSIDERATIONS 17

9.1. General Design 17
9.2. Randomization 17
9.3. Data analysis 17

10. DATA COLLECTION AND QUALITY ASSURANCE 17

10.1. Data Collection Forms 17
10.2. Data Management 17
10.3. Quality Assurance 18
10.3.1. Training 18
10.3.2. Quality Control 18
10.3.3. Protocol Deviations 18

11. PARTICIPANT RIGHTS AND CONFIDENTIALITY 18

11.1. Institutional Review Board (IRB) Review 18
11.2. Consenting Procedure , Informed Consent Forms, and study informants 18
11.3. Participant Confidentiality 19
11.4. Participant Compensation 19
11.5. Study Discontinuation 20

12. PUBLICATION OF RESEARCH FINDINGS 20

13. REFERENCES 21

STUDY TEAM ROSTER

Ihab Hajjar, MD, MS (Principal Investigator)

Section of Geriatrics and Gerontology

Department of Medicine and Neurology

Emory University

ihajjar@emory.com

Allan Levey, MD, PhD (Co-investigator, Neurologist)

Department of Neurology

Emory University

ALEVEY@emory.edu

Arshed Ali Quyyumi, MD (Co-investigator, Cardiology)

Division of Cardiology

Department of Medicine

Emory University

aquyyum@emory.edu

Deqiang Qiu, PhD (Co-investigator, MRI expert)

Division of Magnetic Resonance Research

Department of Radiology

Emory University

deqiang.qiu@emory.edu

Felicia Goldstein, PhD (Co-investigator, Neuropsychologist)

Department of Neurology

Emory University

fgoldst@emory.edu

Jonathan Nye, PhD (co-investigator, Medical Physicist)

Department of Radiology

Emory University

jnye@emory.edu

Mark M. Goodman, PhD (**co-investigator, Radiochemist)**

Department of Radiology

Emory University

mgoodma@emory.edu

John Hanfelt, PhD (Co-investigator; Statistician)

Department of Biostatistics and Bioinformatics, Rollins School of Public Health, Emory University,

Atlanta, GA Atlanta, GA 30322-4201

jhanfel@emory.edu

Nicholas T. Seyfried (Co-investigator, Neuroproteomics)

Director, Emory NINDS-Neuroscience Proteomics Core Facility

Emory University

nseyfri@emory.edu

PARTICIPATING STUDY SITES

Wesley Woods Health Center

Emory University Hospital Clinical Research Network

Executive Park Medical Campus

PRÉCIS

Study Title

Candesartan's Effects on Alzheimer's Disease And Related Biomarkers

Acronym

CEDAR

Objectives

Our *Primary objective* is to determine the safety and dose-response for target engagement of the treatment with escalating doses of candesartan in those with mild cognitive impairment (MCI) and Alzheimer's disease (AD) based on cerebrospinal fluid levels of amyloid and tau.

Our *secondary objectives* are to assess the efficacy of 1 year treatment of candesartan on biomarkers of vascular function, neurodegeneration, and neuro-inflammation; to assess the efficacy of 1 year treatment with candesartan on amyloid imaging; and to estimate the effect size of candesartan on cognitive and functional measures in MCI and AD.

Design and Outcomes

This is a double-blind placebo-control randomized clinical trial that compares candesartan to placebo in individuals with MCI who also have positive AD biomarkers. Our measures include cognitive function, cerebral perfusion and reserve, markers of vascular brain damage, atherosclerosis, arterial stiffness, vascular inflammation and endothelial function. Amyloid and Tau PET imaging will be collected on a subgroup of this trial.

Interventions and Duration

The intervention includes candesartan (8, 16, or 32 mg) or placebo. The duration of the study is 1 year.

Sample Size and Population

Our target population includes subjects who are 50 years or older who are not receiving antihypertensive medications with MCI and AD. Our sample size is 72 for this trial. All 72 subjects will be invited to participate in the additional PET study.

SCHEDULE OF PROCEDURES FOR THE DURTAION OF THE STUDY

Phase:	SCREEN	BASELINE		TITRATION	FOLLOW-UP			
**Number of visits**	1-2	1-3		2	4			
**Months**	-1	0		1-2	3	6	9	12
**Informed Consent**	X							
**Cognitive screening**	X							
**Screening labs**	X*							
**Amyloid Positivity to assess eligibility**	X**							
**CSF for molecular analysis**	X***	X***						X
**Weight/Height**		X		X	X	X	X	X
**Blood Pressure**	X	X		X	X	X	X	X
**Safety labs**				X[#]	X[#]	X[^]	X[#]	X[^]
**Brain MRI**		X	RANDOMIZATION					X
**In-vivo Aβ and Tau PET scan**	X**	X**						X
**Vascular function measures**		X						X
**Cognitive Battery**		X				X		X
**Functional battery (IADL, SPPB)**		X						X
**RAS biomarkers (blood and CSF)**		X				X		X
**APOE genotype**		X						
**Retinal scan and blood draw******								
**Inflammatory/molecular biomarkers (proteomics /metabolomics)**	X[$]	X[$]						X
**Time (in minutes)**	60-120	150-210		30	30	75	30	150-210

*: Screening labs include: CBC, CMP (BUN, CR, electrolytes, liver function), B12, TSH and PT/INR

**: Amyloid positivity can be assessed by either LP (CSF levels of amyloid) or PIB-PET. We will routinely use LP as the default method to determine Amyloid positivity however, in some instances e.g. a failed LP or delayed CSF results due to lab issues then we will use a screening PIB-PET instead. If a PIB-PET was done at screening, it WILL NOT be repeated at baseline. Subjects will only have Tau-Pet at baseline.

***: If subject has had an LP within the 6 months prior to their screening/baseline visit and samples are available for assessing eligibility and for measuring CSF outcomes then no LP will be done at screening. If CSF is only available for eligibility assessment (e.g. from PIB-PET/ clinical LP/assessment/ another research study and subject willing to share these samples) then an LP will be done to obtain CSF for the outcome measures (such

as molecular analysis). This LP can be done at baseline instead of screening (since eligibility would have been already established).
****: Retinal scan +/- 10 cc blood draw are optional and can be done at any visit or after the completion of the study.
#: Safety labs at titration, 3 and 9 months include BMP (BUN, CR, electrolytes)
^: Safety labs at 6 months and 12 months include BMP (BUN, CR, electrolytes) + CBC. If subject started taking any blood thinners during the study, PT/INR will be repeated before LP at 12 months.
$: Samples for inflammatory/molecular biomarkers will be drawn either at screening or at baseline visits.

1. STUDY OBJECTIVES

Our *Primary objective* is to determine the safety and dose-response of target engagement for treatment with escalating doses of candesartan in those with mild cognitive impairment (MCI) and Alzheimer's disease (AD).

Our *secondary objectives* are to assess the efficacy of 1 year treatment of candesartan on biomarkers of vascular function, neurodegeneration, and neuro-inflammation; to assess the efficacy of 1 year treatment with candesartan on amyloid imaging; and to estimate the effect size of Candesartan on cognitive and functional measures in MCI and AD.

2. BACKGROUND AND RATIONALE

Available treatments for AD do not address novel neurovascular and inflammatory pathways. Prior research has documented that the antihypertensive angiotensin receptor blockers (ARBs), in general, and candesartan, in particular, may have a positive effect on both AD biomarkers and clinical manifestations.

2.1. DISEASE TARGETS FOR ARBS IN AD

AD is a multifactorial and a multisystem disease. ARBs target vascular, inflammatory, and amyloid pathways involved in AD initiation or progression shown in **Figure 1**. At the neurovascular level, ARBs improve cerebral perfusion and restore neurovascular coupling and microvascular function.[1-3] Both MCI and AD individuals exhibit significant microvascular dysfunction as reflected by declines in vasodilator response to CO_2 (Vasoreactivity).[4] Our work suggests that in MCI, RAS activation leads to lower vasoreactivity, and ARBs may improve this measure which translates into improved cognition.[1,5] Peripheral vascular markers including increased arterial stiffness (which correlates with amyloid deposition in the brain[6,7]) and decreased microvascular regeneration capacity reflected by circulating endothelial-related progenitor cells (EPC)[8] are both observed in AD. EPC's have a potential role in repairing the blood brain barrier (BBB) endothelium[9,10] by differentiating into BBB like-cells[11,12] and improving neurovascular healing.[13] EPC are decreased in AD patients [8] and correlate positively with cognitive function.[8] Ang II (via AT1) leads to smooth muscle hypertrophy[14] and release of endothelial-related inflammatory mediators,[15] resulting in increased stiffness.[16] Aβ[17] and Ang II[18] reduce EPC counts and function leading to apoptosis, and ARBs may inhibit these effects in animal models.[19] ARBs also inhibit Aβ induced neurovascular impairments, independently of BP.[20-22] Aβ is transported back from the blood to the brain by receptors for advanced glycation end products (RAGE) on the endothelium.[23] Ang II up-regulates RAGE and increases BBB permeability by increasing oxidative stress[24] and disrupting tight junctions.[25,26] ARBs inhibit the expression of RAGE[27] and restore BBB integrity by "healing" its endothelium. Ang II also increases and ARBs inhibit glycogen synthase kinase 3β (GSK3β) activity.[28] GSK3β increases tau phosphorylation and synaptic defects and is initiated in the earliest stages of clinical AD.[29-31] ARBs hence may decrease p-tau levels as suggested by a recent rat experiment. [32] This multi-target characteristic of ARBs increases the likelihood of producing a disease-modifying effect, rather than a symptomatic effect as provided by currently approved medications.

Figure 1: Vascular, inflammatory, and amyloid Targets for ARBs

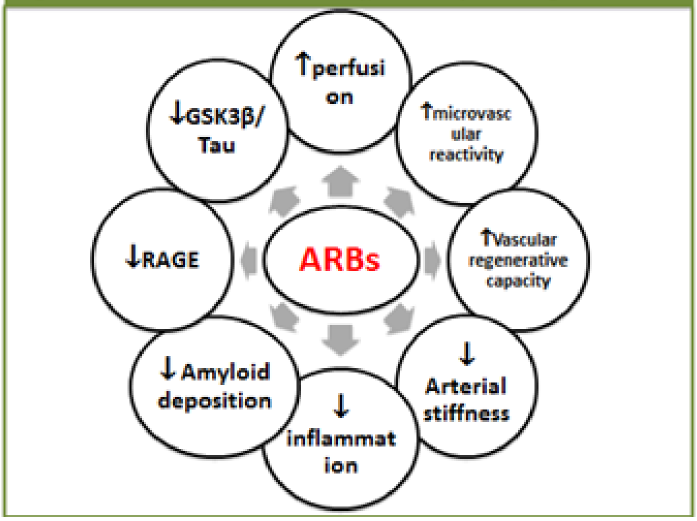

2.2. SAFETY OF ARBS IN NON-HYPERTENSIVE INDIVIDUALS

Prior studies in non-hypertensive individuals with normal cognition have shown that it has a good safety profile with respect to adverse events and hypotension. In a non-hypertensive sample, Losartan (at equipotent candesartan doses; n=142 non-hypertension) had similar BP declines (4.7/3 mm Hg) compared to placebo.[33] (**Table-1**) In the Diabetic Retinopathy Candesartan Trial Program [n=3,326, follow-up=5 years, pretreatment BP: 115-118/70-75 mm Hg], systolic blood pressure (SBP) reductions were in the range of 2.6-3.6 mm Hg in the candesartan arm and 2.5-2.7 in the placebo arm with no diastolic blood pressure (DBP) changes.[34] These declines were not associated with hypotensive symptoms.

Table 1: Pre-and post-treatment blood pressures in 142 non-hypertensive individuals

	Week 0	Week5 (50 mg)	Week10 (100 mg)
**Losartan**	135.9/78.8	132.4/76.8	131.3/75.8
**Placebo**	138.3/80.3	136.1/79.7	138.4/80.7

2.3. STUDY RATIONALE

In the brain, Angiotensin II (Ang II) binds AT1 and AT2 receptors. AT1 activation increases oxidative stress,[35] endothelial dysfunction, vasoconstriction and hypoperfusion, cholinergic depletion, neuro-degeneration, and memory loss.[36-39] AT2 improves endothelial function, increases cerebral perfusion,[40] decreases superoxide production,[40] promotes neuronal cell differentiation,[41-48] and facilitates memory functions.[49,50] Blocking the effect of Ang II, a crucial mediator of neuro-vascular injury, using ARBs may address shortcomings of current treatments. ARBs affect multiple systems involved in AD: vascular function, blood pressure (BP), inflammation, and the amyloid cycle.[see review[51]]

3. STUDY DESIGN

This is a **PHASE II** 1-year double-blind placebo-controlled randomized clinical trial where participants will be treated with candesartan (escalating doses: 8, 16 to 32 mg) or matched placebo.

4. SELECTION AND ENROLLMENT OF PARTICIPANTS

4.1. INCLUSION CRITERIA

(1) Age: 50 years or older;

(2) MCI will be defined as:

(i) Subjective memory concern;

(ii) Abnormal memory function documented using the Logical Memory subscale (Delayed Paragraph Recall, Paragraph A only) from the Wechsler Memory Scale-Revised (the maximum score is 25): [<11 for 16 or more years of education; <9 for 8-15 years of education; <6 for <7 years of education];

(iii) Montreal Cognitive Assessment (MoCA) < 26;

(iv) Clinical Dementia Rating scale /Memory sum Box score=0.5.

(v) General functional performance sufficiently preserved (Functional Assessment Questionnaire<9);

(3) Amyloid positivity: This will be determined by measuring the amyloid content in the brain. This can be determined by either CSF amyloid level or an amyloid scan (PIB-PET). We will plan to use CSF levels of amyloid as our default method. However, if we are not able to determine eligibility using CSF levels then a PIB-Pet will be performed to assess amyloid positivity.

In instances where the diagnosis of MCI is not clear, a review of the subject cognitive and non-cognitive data will be performed by the study physicians or neuropsychologists and a diagnosis of MCI will be based on the clinical judgment of the study physicians or neuropsychologists.

4.2. EXCLUSION CRITERIA

(1) Intolerance to ARBs;

(2) Current use of ARBs or ACEI (use of antihypertensive medications other than ACEI or ARBs for other indications is allowed);

(3) Current diagnosis of hypertension or current use of antihypertensive medication that is prescribed specifically for hypertensive therapy;

(4) SBP less than 110 or DBP less than 40 mm Hg;

(5) Renal disease (Creatinine >2.0 mg/dl), hyperkalemia (K>5.5 meq/dl), platelets<50,000/µl, or INR>1.9;

(6) Active medical or psychiatric diseases that in the judgment of the investigator would affect the safety of the subject or scientific integrity of the study e.g. if the patient has hypothyroidism or low vitamin B12 that is contributing to the subject's cognitive impairment;

(7) Uncontrolled congestive heart failure reflected by poor exercise tolerance and shortness of breath;

(8) History of stroke in the past 3 years;

(9) Inability to have MRI (eg metal implants or cardiac pacemaker) with an exception for those who cannot have an MRI, if all other parts of the study are obtained successfully they may still be enrolled in the study, or cognitive assessment or inability to assess amyloid positivity (no LP and no amyloid scan);

(10) History of increased intracranial pressure (ICP) or bleeding diathesis (from disease states or from use of anticoagulants such as warfarin, heparin and related products, Rivaroxaban or Xarelto, Apixaban or Eliquis, Edoxaban or Savaysa, Dabigatraban or Pradaxa);

(11) Women of childbearing potential (non-menopausal);

(12) In those who are unable to demonstrate that they understood the details of the study (ie lack of decisional-capacity to consent), a study partner/surrogate who can sign on their behalf will be required, otherwise they will be excluded;

(13) Current use of Lithium, as Candesartan may increase lithium concentration to toxic levels.

4.3. STUDY RECRUITMENT

Potential participants will be identified through community activities, health fairs, and patient referrals. Below we describe our recruitment venues:

(1) The Emory Alzheimer's Disease Research Center (ADRC) Clinical Core registry of research participants will be utilized for recruitment. Participants have agreed to be referred to other studies.

(2) Community-based recruitment: Community education sessions in local neighborhoods e.g. churches or barbershops will be conducted and attendees will be invited to participate in this study.

(3) MindMate App recruitment: MindMate users have the opportunity to first express their interest in clinical studies within the 'Research' section of the app. MindMate can also reach out to users once they have consistently utilized the app, usually after 3-4 weeks, & ask the user if they would like to receive further

information when a matching clinical study is available in their area. All users that are interested in participating in a clinical study must then go through a multi-stage opt-in & consent process. After MindMate matches a user to an open clinical trial, they automatically ask if they're happy to be contacted by a researcher to learn more about an open study they might qualify for.

(4) Physician recruitment: Local physicians (primary care or specialty physicians) will be informed of the study and its criteria and provided information about referral to the study personnel. In addition, flyers for the study will be posted at outpatient areas in the local medical facilities.

5. STUDY INTERVENTIONS

5.1. INTERVENTIONS, ADMINISTRATION, AND DURATION

Participants will be randomized to either candesartan or placebo in a 1:1 fashion. All participants will be initiated on 8 mg. The dose will be increased in 2 week increments to 16 mg and 32 mg as long as: SBP>110 mm Hg, DBP>40 mm Hg and participant reports no symptoms of hypotension (dizziness or weakness). The highest achievable dose will be the Maximal Tolerated Dose (MDT) and will be prescribed for the duration of the study. The cut-off of 110 mm Hg for SBP is based on the evidence that a decrease of ≤5 mm Hg in SBP is expected in non-hypertensive individuals. Hence, a cutoff of 110 mm Hg in SBP decreases the risk of developing hypotension (defined as <100 mm Hg in SBP). Treatment will be provided in similar capsules format to be taken once a day orally. Investigators, study personnel and participants will be blinded for drug assignment. Participants will be treated for 1 year.

5.2. HANDLING OF STUDY INTERVENTIONS

The drugs will be stored in the medication room in the Investigational Drug Service (IDS) Pharmacy. The location of the IDS pharmacy is The Emory Clinic Bldg. A, Suite 1200, 1365 Clifton Road, NE, Atlanta, Georgia 30322. Access to the med room is limited to the IDS Pharmacists via a badge swipe. Accountability records are maintained for all investigational product (IP). A courier will deliver prescriptions to study location to be given to subjects or study staff will be able to pick up prescriptions.

5.3. CONCOMITANT INTERVENTION

5.3.1. ALLOWED INTERVENTIONS

Participants will continue to receive their usual care from their regular primary care physicians. In the event a participant becomes hypertensive during the trial, the participant will be referred to the primary care provider for further hypertension management. Participants will be allowed to use any class of antihypertensive medication except another ARB, an ACEI, or a renin inhibitor. The PMD will be notified of the potential that the subject might be receiving candesartan to avoid this overlap.

5.3.2. PROHIBITED INTERVENTIONS

Additions of an ARB, ACEI, or renin inhibitors are prohibited during the study period. Participating in another clinical trial or study is prohibited.

5.4. ADHERENCE ASSESSMENT

Participants will be asked to bring their study medication bottles to the study center at each visit. Medication compliance will be assessed using pill count during the titration and follow up periods. We will define a compliance rate for a time period, t, as the ratio of: (the used number of pill prescribed for the number of days t - number of pills remaining or unused for the time t / number of pills prescribed for time t) multiplied by 100.

6. STUDY PROCEDURES AND MEASURES

6.1. QUESTIONNAIRES, LIFESTYLE AND ANTHROPOMETRIC MEASURES

Study interviews will be conducted in English and include the following:

(1) Demographic, social, stress, physical activity data and medical history data, as well as a medication inventory will be collected. All participants are asked to bring all their prescribed medication bottles.

(2) Instrumental activities of daily living (IADL) scale .[52]

(3) Weight and height (stadiometer to measure height with the subjects standing and balance beam scale to measure weight without shoes).

(4) Short Physical Performance Battery [SPPB: ability to stand with the feet side-by-side, semi-tandem, and tandem, time to walk 8 feet (measured twice), and time to rise from a chair and return to the seated position 5 times).[53]

6.2. BLOOD PRESSURE MEASUREMENT

Office blood pressure will be measured according to the American Heart Association guidelines: sitting position, rested for 5 minutes, appropriate cuff size (covering 60% of upper arm length and 80% of arm circumference), correct cuff placement (1-2 inches above brachial pulse on bare arm). Measurement will be done manually using the bell of the stethoscope or using a validated and calibrated automatic machine. Blood pressure will be measured in both arms. The arm with the higher blood pressure will be used throughout the study. We will obtain 2 seated readings followed by 2 standing blood pressure measurements at 1 and 3 minutes during each visit.

6.3. NEUROPSYCHOLOGICAL ASSESSMENT:

(i) *Clinical Dementia Rating- (CDR)*. The CDR rates each of the six general domains (or boxes) involving memory, orientation, judgment and problem-solving, community affairs, home and hobbies, and personal care, and a global rating is then generated, ranging from 0-no impairment to 3-severe impairment. A Study informant or study partner will be questioned either by phone or in person to assist with the CDR.

(ii) EXecutive Abilities Measures and Instruments for Neurobehavioral Evaluation and Research or "EXAMINER"[54]: This is a test battery that reliably and validly assesses executive function in clinical trials: working memory, inhibition, set shifting, and fluency. The EXAMINER battery has excellent psychometric properties with test-retest reliability of over 0.9 and correlates by over 0.60 with an informant-based measure of day-to-day executive functioning, the Frontal Systems Behavior Scale. The parts of EXAMINER that we selected include:

> 1) Flanker task (inhibition) which involves responding to a central stimulus while ignoring flanking stimuli that are either compatible or incompatible with the central stimulus.[55]
>
> 2) Set-shifting, a measure of mental flexibility assessing the subject's ability to attend to the specific attributes of compound stimuli, and to shift that attention when required.[56]
>
> 3) Spatial 1-Back test assesses spatial working memory and
>
> 4) Dot Counting test assesses verbal working memory.[57]
>
> 5) Verbal Fluency will be tested using a List Generation test which require the participant to generate words beginning with a specific letter, and category fluency in which the participant generates words from a specified category (e.g., animals, fruits).[58]

(iii) To assess additional cognitive domains/mood, we will use these tests included in the National Alzheimer's Coordinating Center Uniform Data Set.[59,60]

1) Hopkins Verbal Learning Test will be used to assess memory domains.[61]

2) Digit Span Test (DST) is a brief task that assesses attention.[62,63]

3) Boston Naming Test assesses language by measuring ability of naming a visual confrontation drawing (15 items).[64,65]

4) Trail Making Test: will be used as an additional measure of executive function.

5) Center for Epidemiologic Studies Depression Scale (CESD),[66] and consists of 20 items, each scored from 0 to 3 points and higher scores indicate greater depressive symptoms.

(iv) Voice recording to assess speech changes related to cognitive function may be collected at one or more visits. These will include a recording of the cognitive tests, free speech, speech in response to questions, and speech in response to specific cognitive tasks such as counting, fluency and/or a picture description. All voice recording files will not contain personal identifiers or PHI. They will be saved under the subject deidentified study number.

6.4. CEREBROSPINAL FLUID (CSF) COLLECTION

Participant will have CSF acquired via lumbar puncture (LP). In some instances, subjects would have had an LP as part of clinical evaluations or other research studies. If we are able to determine eligibility from the prior LP (within 6 months of the screening visit) and samples are available for the molecular analysis, then screening LP will not be necessary. Otherwise an LP will be done at screening. All CSF samples will be collected after at least 6-hour fast from the last meal. CSF will be collected using a 24-g Sprotte atraumatic spinal needles. This needle decreases the frequency of post LP headaches to < 1%.[67] All CSF collection is completed according to guidelines put forth in the "Biospecimens Best Practice Guidelines for the ADCs" published by the National Alzheimer's Coordinating Center (NACC) and available on their website. Approximately 30-45 ml of CSF will be collected using sterile polypropylene collection tubes. The samples will be immediately divided into aliquots and transferred for storage in a freezer for future assays. In addition to routine assessment (cells, proteins, albumin, glucose), Aβ1-42, t-tau, and p-tau181p will be measured using the multiplex xMAP Luminex platform (Luminex Corp, Austin, TX) with Innogenetics (INNO-BIA AlzBio3; Ghent, Belgium; for research use–only reagents) immunoassay kit–based reagents[68] at the local ADRC core laboratory.

6.5. BRAIN MRI AND PET IMAGING PROTOCOLS

MRI protocols are performed in 50-70 minutes and will be conducted at The Center for Systems Imaging (CSI). The MRI protocol includes:

(i) Structural MRI and WMH: High-resolution anatomical images will be acquired using a 3D-Fast Spoiled Gradient Recalled Echo (FSPGR) Sequence. WMH regions will be identified by a 3D T2 Fluid Attenuated Inversion Recovery (FLAIR) Fast Spin Echo sequence.

(ii) ASL-MRI: ASL images will be acquired using a custom 3D stack of interleaved spirals fast spin echo sequence and will be averaged in order to improve the signal-to-noise ratio. In addition to resting perfusion, scans will be taken during normocapnia (2 minute scan) and hypercapnia (breathe a mixture of 8% CO2 for 2 minute scan). The following metrics will be calculated: resting perfusion, Vasoreactivity (VR)=slope of the regression between cerebral perfusion and end-tidal CO2, in ml/100g/min per mm of Hg during normal and hypercapnia.

(iii) Diffusion tensor imaging: DTI is highly sensitive to detect structural changes over a short time, less than 1 year, as compared to traditional MRI.[69]

(iv) Resting state functional MRI (rs-fMRI): Resting state fMRI will be used to assess "functional connectivity" between brain regions.[70] Functional connectivity has a key role in important complex cognitive processes.[70] It

focuses on spontaneous, rather than task-induced, low frequency (<0.1 Hz) fluctuations in the blood oxygenation level-dependent (BOLD) signals.

(v) _In-vivo Aβ- and Tau PET imaging_: This will be performed on the original sample (n=72). Participants will be selected consecutively for the original study until up to a total sample of 72 complete the PET studies (36 per group). In-vivo amyloid imaging will be conducted using N-methyl-[^{11}C] 2-(4'-methylaminophenyl)-6-hydroxybenzothiazole also termed [^{11}C] Pittsburgh Compound B (PiB),[71] following ADNI protocol.[72] PET images (20 minutes; 4 × 5 minute frames) will be acquired approximately 50 minutes after intravenous administration of 15±1.5 mCi of [^{11}C] PiB. Images will be analyzed as recently recommended.[73] In certain cases (see eligibility section) PiB Pet can be performed at screening to determine amyloid positivity. *In-vivo Tau-PET imaging* will be conducted using ^{18}F-labeled benzimidazole pyrimidine, 7-(6-[^{18}F]fluoropyridin-3-yl)-5H-pyrido[4,3-b]indole ([^{18}F]T807) which has been shown to have selectively high affinity to Tau; and T807 PET scans are now available to measure in-vivo Tau content in patients with MCI and AD.[74-78]

6.6. VASCULAR MEASURES

Arterial stiffness and microvascular function: Indices of arterial stiffness and wave reflections will be estimated in the supine position using the Sphygmocor device (Atcor Medical, Australia), which records sequential high-quality pressure waveforms at peripheral pulse sites using a high-fidelity tonometer. Pulse-wave velocity (PWV) measured between carotid and femoral arteries is a regional assessment of aortic stiffness and is the gold standard index of arterial stiffness. Digital pulse amplitude tonometry (PAT) will be used to measure pulse volume amplitude (PVA) in the tip of the index finger, with participants resting in the supine position in a quiet, temperature-controlled environment and during reactive hyperemia, which will be elicited by the release of an upper arm blood pressure cuff inflated to supra-systolic pressure for 5 minutes. The Endo-PAT (Itamar-Medical, Israel) will be used to measure PAT. The reactive hyperemia index (RHI) is a marker of endothelial function.

Blood pressure and Ultrasound: A brachial BP will be measured twice while seated and after 1 and 3 minutes standing according to the AHA guidelines.[94] High-resolution B-mode ultrasound of the right common carotid artery using a 7.5-MHz linear-array will be performed to measure carotid intima-media thickness (IMT).[95]

6.7. PROTEOMICS AND INFLAMMATORY MARKERS

We will conduct proteomic and metabolomics studies of the CSF and blood samples (screening/baseline & 12 months) at the Emory Neuroscience NINDS Core Facilities (ENNCF) and the Emory Metabolomics core. We will use a targeted proteomic approach with selected reaction monitoring (SRM).[82] Our set of proteins in the targeted proteomic analysis are enriched with the inflammatory/oxidative stress pathways: adhesion molecule,[83] cytokines (see Hu et al[84] for full list), oxidative stress (F2-isoprostanes),[85,86] and BBB integrity proteins (astrocytic protein S100β, neuron-specific enolase (NSE) and RAGE).[87-89]

6.8. RENIN ANGIOTENSIN SYSTEM ACTIVITY

RAS activity will be assessed using a comprehensive analysis of different angiotensin peptides measured simultaneously in a single sample by a highly selective and sensitive mass spectrometry method described previously.[90] We will use this to assess the degree of blockade of AT1 and activation of AT2.

6.9. BLOOD CHEMISTRIES, BLOOD COUNT, AND APO-E

Blood will be drawn at various visits. Please refer to the table for specific tests done at each visit.

6.10. BLOOD AND CSF BANKING

We will collect blood and CSF samples for future research. Vials containing blood samples will be labeled with subject ID numbers, name of the study, date collected and barcoded and stored at the cryogenic storage facility

until time of processing. The label will include the study name, study visit number, contents of each collection vial, and the date of collection. The samples will be kept until the samples are exhausted. Future study results obtained from these samples may not be reported to subjects. For those who agree to have their retina scanned, an additional (10 cc) of blood will be drawn during or close to the retinal scan visit.

6.11. RETINAL AMYLOID SCAN

We will perform retinal imaging on the study subjects to test the utility and suitability of retina imaging for detecting AD markers. This is a non-invasive procedure that requires dilation of the eyes and obtaining photographs of the retina. Retinal scans can be done during or after the participant has completed the study. Those already completed will be re-contacted for this procedure.

6.12. SCHEDULE OF EVALUATIONS

6.12.1. SCREENING

Individuals who express an interest will have a 2-tier screening process. Individuals meeting criteria of a clinical diagnosis of MCI will undergo further screening. Those who are eligible, i.e. have MCI will undergo a clinical evaluation (clinician interview, review of prior imaging, review of prior CSF analysis). If no clinical data is available to confirm AD biomarkers in the CSF within the last 24 months, an LP will be performed. This will be scheduled on a separate visit or on the same day if safety labs are available on that day.

6.12.2. ENROLLMENT, BASELINE, AND FOLLOW-UP

Once eligible, baseline evaluation will be completed. An LP will be done if not done during the screening visit. Once baseline data are collected, participants will be randomized into candesartan or placebo and seen every two weeks where study dose will be increased to 16 mg and 32 mg until MDT is achieved. They will be seen afterwards every three months until the study ends. All visits will have a +/- three week window except during medication escalation visits where the window will be +/- 5 days. An optional modified phone/virtual visit will be completed as needed for any visit to collect data that can be reasonably collected by phone. This change is in response to the COVID-19 stay home orders and in-person research activities will resume when normal business operations resume. Study procedures are shown in the **Table above**.

7. SAFETY ASSESSMENTS

7.1. SPECIFICATION OF SAFETY PARAMETERS

(i) The following safety parameters are related to the use of Candesartan: rash, dizziness, cough, weakness, fatigue, headaches, lower extremity edema, constipation, back pain, sore throat, upper respiratory tract infection, rhinitis, pharyngitis and hoarseness. Angioedema, renal failure (Cr>2.5), hyperkalemia (K>5.9 meq/dl), and fainting are also potential complications of candesartan.

(ii) The following safety parameters are related to the conduct of the study procedures: Neuropsychological assessment may be accompanied by anxiety, frustration and overall fatigue. The attachment and removal of a blood pressure cuff, cuffs for venous occlusion, and ultrasound probe on the neck may cause mild discomfort. Brain imaging requires the participant to stay still and lie down for 50-70 minutes, which may cause boredom and minimal reversible back pain. Because of the closed space and noise, undergoing an MRI may be associated with anxiety or panic reactions. We will ask participants to breathe via a mask air richer in CO_2 than normal atmospheric air (5-8%) during the brain MRI procedure. The reported potential side effects may include a feeling of dizziness, faintness, or anxiety during CO_2 inhalation. CO_2 administration will be promptly terminated if any of the aforementioned conditions occur. The vascular ultrasound is safe and only mildly uncomfortable due to

tightness in the arm when the cuff is inflated. We will use ultrasound to painlessly bounce sound beams off of the carotid artery using a pencil-like sensor.

(iii) The following safety parameters are related to the LP procedure: The most common complication of a lumbar puncture is post-LP headache. We use a smaller 24-gauge Sprotte spinal needle. All subjects will be counseled on the signs and symptoms of post-LP headaches and given information on management (acetaminophen, caffeine, rest). Bacterial meningitis and a CSF leak are very rare complications of lumbar punctures. A blood patch is rarely needed to heal CSF leaks. All subjects will be instructed to promptly contact us if the headache persists beyond 3 days and if a blood patch is required, the cost of the patch will be covered by the funds supporting this project.

(iv) Potential risks of the study PET imaging are as follows: There may be discomfort, bruising, bleeding or infection at the site of the intravenous puncture. The radiation exposure is within limits acceptable to the Emory University Radioactive Drug Research Committee. Systemic toxic effects attributable to single dose T807 or PiB have not been encountered. Both ligands are rapidly cleared from the plasma and their binding to their target is reversible. Toxicity remains a possibility and patients will be monitored for such events during the scan and at follow-up.

(v) Potential risks of the retinal amyloid scan are as follows: Adverse reactions to dilation of the eyes may include discomfort, irritation, or other reactions similar to pupillary dilation experienced during a visit to the eye doctor. Increased sensitivity to bright lights may be experienced. We will provide disposable sunglasses to counteract these effects. Participants will be monitored for adverse effects.

7.2. METHODS FOR ASSESSING AND RECORDING SAFETY PARAMETERS

During the course of the study, any problem reported by the participants will be recorded in the participant's chart (adverse event log), along with the start and stop dates. The problem may be related to the research participation or the drug used in the study (drug reaction). All Adverse events will be rated on the following characteristics:

(i) Serious (YES/NO): A serious adverse event (SAE) is defined using the Code of Federal Regulations Title 21 definition as any event that results in any of the following outcomes: Death, a life-threatening adverse event, inpatient hospitalization or prolongation of existing hospitalization, a persistent or significant incapacity or substantial disruption of the ability to conduct normal life functions, or a congenital anomaly/birth defect.

(ii) Related to the research: All adverse events will be judged regarding their relationship to the research participation or the study drug as related, possibly related, probably related, unrelated, or unknown.

(iii) Severity: The severity of the AE will be rated on the following scale mild, moderate, severe, life-threatening, or death.

(iv) Anticipated or unanticipated: We use the Emory IRB definition of an unanticipated event as any event that is "unexpected, not described in the study documents, or if described before, it is now presenting with increased severity, duration, or frequency".

7.3. REPORTING ADVERSE EVENTS AND DEATH

Principal Investigators shall immediately (within 24 hours of the event) report to the Radioactive Drug Research Committee all adverse effects associated with the use of the radioactive drug in the research study. All adverse reactions probably attributable to the use of the radioactive drug in the research study shall be immediately reported (within 24 hours of receiving the initial report) by the RDRC to the Food and Drug Administration, Center for Drug Evaluation and Research.

All other adverse events will be assessed for the need for prompt reporting. We follow the guidelines set-forth by the Emory IRB for reporting AE's. Any event that fulfills the following criteria being unanticipated (as defined in section 7.2), probably or possibly related to study participation (drug effect or as a consequence of a study procedure), and involving risk for participants or others is considered an unanticipated problem and will be reported to the Emory IRB within 10 days. If an event could be explained by the underlying medical condition, it is not considered related and hence not reportable. Any SAE as defined in **section 7.2** that does not fit all the criteria of being an unanticipated problem will be reported during continuing review. Deaths of participants during the study will be promptly reported to the IRB if related to study participation and reported during continuing if they are not related to study participation. A yearly summary of AE/SAE/Deaths will be presented to the DSMB by the study biostatistician.

7.4. FOLLOW-UP FOR ADVERSE EVENTS

All AE/SAE's will be recorded in the participant chart with start/stop date. The study physician may perform additional evaluations, visits, phone communication, repeat blood testing, or refer to the emergency room or the participant healthcare provider for medical care if necessary. If the event results in intervention discontinuation and/or study withdrawal, the participant will be followed for 2 months post-withdrawal with a monthly phone call. They will also be invited to perform the final visit evaluation including all study procedures planned for that visit if the participant has been in the study more than 5 month post baseline evaluation.

Follow-up on reported symptoms of hypotension or a BP<100/40 mm Hg: When a subject notifies the study team that they have either symptoms suggestive of hypotension (dizziness, weakness, near syncope, or syncope) or that their blood pressure is less than 100/40 mm Hg, the study physician will review the incident and determine if the subject is safe to continue in the study. The physician may discontinue the study medication immediately, elect to lower the dose of the study medication, ask subject to present to the study center for a safety evaluation, and/or monitor the subject blood pressure. The subject may continue in the study if blood pressure is >100/40 mm Hg on the lower dose of the study medication and had resolution of symptoms. In those on 16 mg and 32 mg, the doses will be decreased to the next lower dose. Those on 8 mg of candesartan, a trial of every other day 8 mg of candesartan will be attempted prior to discontinuation.

7.5. INCIDENTAL FINDINGS

Incidental findings (lab testing, neuroimaging or vascular testing) identified during the conduct of the study that do not require immediate medical care will be reported to the subject with instructions to seek medical care from the participant's healthcare provider. Those with incidental findings requiring urgent care will be referred to an emergency room in a facility of their choosing.

8. INTERVENTION DISCONTINUATION

A subject may choose to withdraw from the study for any reason. The investigators may also request that the subject withdraw from the study, for safety or other reasons. The criteria for discontinuing a subject's participation include:

(1) the subject's request,

(2) adverse events that requires un-blinding or necessitate stopping the study drugs,

(3) persistent (>2 episodes on 2 separate days) of symptomatic hypotension or a blood pressure <100/40 mm Hg on 2 separate occasions despite adjusting study medication to the lowest dose (see section on follow-up on AE 7.4).(4) new clinically evident stroke or myocardial infarction or other event that limits ability to complete the study procedures

(4) inability to participate due to relocation or other personal reasons,

(5) renal failure (increase in serum Creatinine above 2.5 mg/dl) or hyperkalemia (greater than 5.9 meq)

9. STATISTICAL CONSIDERATIONS

9.1. GENERAL DESIGN

The study is a 2-arm double-blinded RCT. The statistical analysis will follow the intention-to-treat approach. Intention-to-treat (ITT) and per-protocol analyses will be performed.

9.2. RANDOMIZATION

The study blinded biostatistician will provide oversight of randomization fidelity and blinding. Randomization will be stratified by use of cholinesterase inhibitors or Memantine (yes vs no) using a computerized random number generator (SAS, V9.4). Only the pharmacy will have access to the randomization lists. As the pharmacist is notified that an individual subject is eligible for randomization, the pharmacist prepares the appropriate blinded study product. The pharmacist will enter the subject ID, date of product randomization, and the unique allocation sequence number on a web-based data form.

9.3. DATA ANALYSIS

Table 2 provides a list of main outcomes.

**Table 2: Independent, main outcome and other variables to be used in the analyses**	
**Independent:**	Group (Candesartan vs placebo)
**Safety outcome:**	hypotensive episodes, symptoms (dizziness, weakness, cough) , creatinine >2.5 mg/dl , K>5.9 mEq/dl, number of discontinuations of candesartan, AE, SAE, Deaths
**CSF outcomes:**	CSF tau, inflammatory proteins
**Vascular outcomes:**	PWV, augmentation index
**Imaging outcomes:**	Perfusion, hippocampal volume, vasoreactivity, Global SUVR (PiB and T807)
**Cognitive outcomes:**	CDR, EXAMINER, HVLT, TMT (B and B-A)

Baseline characteristics including demographic, APOe, social and cognitive measures will be compared between the 2 groups to assess randomization fidelity. The list of the main variables is shown in Table 3. We will compare the change in our outcomes over the study period between the treatment and placebo arms using Mixed Models. For the dose response analysis, the dose vs RAS activity will be plotted to explore the degree of modulation for each Ang peptide at the various doses.

10. DATA COLLECTION AND QUALITY ASSURANCE

10.1. DATA COLLECTION FORMS

Data collected during interviews and exams will be documented on trial-specific data forms. Neuroimaging and ultrasound data will be saved in digital formats on a HIPAA-compliant server.

10.2. DATA MANAGEMENT

Once a subject is enrolled into the study, he/she will be assigned a unique identifier number and be referred to by initials and the study number only. Only research team members will have access to the files. Data will be entered on a web-based secure trial data system; the trial database will include for all variables an electronic data audit of data edits (who, when, and why). A data query report (including missing, out of range, and logic

checks) will be generated by the trial statistician. The investigators will keep subjects' medical records private as far as the law allows. The IRB and officials of the sponsor/funding agency will have access to these records as needed within legal guidelines. If study results are published in journals or presented at meetings, we will not use the subjects' names.

10.3. QUALITY ASSURANCE

10.3.1. TRAINING

Research personnel will be trained by the PI and co-investigators. The process of training on data forms completion, neuropsychological assessment and subject evaluation will be documented in a training log for each study personnel.

10.3.2. QUALITY CONTROL

To assess protocol compliance and quality of data collected, the PI along with another investigator will randomly and on intervals review data obtained. In addition, assessment of personnel competencies in obtaining data will be performed.

10.3.3. PROTOCOL DEVIATIONS

Every attempt will be exercised to maintain compliance with the approved study protocol. In the event when a deviation is noted, the Emory IRB will be notified as required by IRB Policies & Procedures only if the deviation affects the rights or welfare of subjects, the safety of subjects, the willingness of subjects to continue with study participation, or the integrity of the research data. The PI or designated personnel will conduct an investigation about the setting, reasons, and potential remedies that need to be instituted to rectify the deviation and prevent future similar instances.

11. PARTICIPANT RIGHTS AND CONFIDENTIALITY

11.1. INSTITUTIONAL REVIEW BOARD (IRB) REVIEW

This protocol and the informed consent document and any subsequent modifications will be submitted and reviewed by the Emory IRB.

11.2. CONSENTING PROCEDURE, INFORMED CONSENT FORMS, AND STUDY INFORMANTS

A signed consent form will be obtained from each participant or the participant's legally authorized representative. A single informed consent form will describe both the screening and study procedures. The consent form will describe the purpose of the study, the procedures to be followed, and the risks and benefits of participation. A copy will be given to each participant and this fact will be documented in the participant's study record.

During the consenting process, an assessment for decisional capacity to participate in clinical research will be performed. This is conducted by asking the subject a set of study-specific questions. A validated brief instrument for decisional capacity assessment will be administered:[91] The University of California, San Diego Brief Assessment of Capacity to Consent (UBACC) instrument is a 10 item questionnaire that asks the participant about key areas of the informed consent. A score greater than 14.5 correlates with a score of greater than 16 on the MacArthur Competency Assessment Tool for Clinical Research (MacCAT-CR).[91] MacCAT-CR is the most validated instrument in assessing decisional capacity in clinical research[92] and a score greater than 16 has been traditionally considered adequate in prior NIH studies.[93]

Participants who are unable to answer the capacity assessment questions correctly (UBACC score <14.5) may still qualify for the study but will need to have a surrogate and a study informant. The definition of the surrogate

will be consistent with the intent of the Common Rule (45 CFR 46, Subpart A).The following are, in order, possible surrogates:

(1) The person's agent designated by an advance health care directive.

(2) The conservator or guardian of the person having the authority to make healthcare decisions for the person.

(3) The spouse of the person.

(4) The domestic partner of the person as defined in Section 297 of the Family Code

(5) An adult son or daughter of the person.

(6) A custodial parent of the person.

(7) Any adult brother or sister of the person.

(8) Any adult grandchild of the person.

(9) An available adult relative with the closest degree of kinship to the person.

When there are two or more available persons who are in different orders of priority pursuant to subdivision (c), refusal to consent by a person who is a higher priority surrogate shall not be superseded by the consent of a person who is a lower priority surrogate. That surrogate will need to consent to the study. If either party refuses, we do not enroll the subject. To ensure both the participant and the proxy understand the study protocol, we will ask subjects or legal next of kin to explain in their own words the nature of the study and the procedures involved.

The proxy will be considered the study informant if there is contact at least once a month. In some instances such as the lack of adequate contact by the surrogate or the surrogate's inability to complete the tasks of the study informant, the study informant may be a different individual as long as he/she is willing to provide information about the participant, and have contact with the participant for at least once a month (in person or telephone). The study informant will sign the study informed consent. If they are not available in person, the study staff will contact the informant to obtain needed information about the subject cognitive symptoms. In that case, a verbal consent will be obtained.

11.3. PARTICIPANT CONFIDENTIALITY

Only the investigators will have access to information about a particular subject. The subject's primary care physician will only be notified if the subject agrees. To maintain confidentiality, subject data will be referenced by number and stored in locked computer files and cabinets. Identifying information about a subject will not be used during the discussion, presentation, or publication of any research data. Only research team members will have access to the files. Data recorded and stored on the computer will be backed up to a disc and stored with the paper files. Participants will not be given any results of the research procedures unless there is a medical necessity. This will be clearly stated in the informed consent process.

11.4. PARTICIPANT COMPENSATION

Subjects will be compensated with gift cards for their effort and time at the following schedule:

- Screen Visit: $25 + $100 (for lumbar puncture procedure)
- Baseline Visits: $100 (for visit + MRI) + $75 for each PET scan (optional)
- Titration Visits (2): $10
- 3 & 9 Month Follow Up Visits: $25
- 6 Month Follow Up Visit: $50 (cognitive testing, blood draw)

- 12 Month Follow Up Visits: $100 (for visit + MRI) + $75 for each PET scan (optional) + $100 (for lumbar puncture procedure)
- Return Visit for blood redraw: $20 and LP $100 (as needed)
- Retinal Imaging: $25Transportation: Up to $30 for gas reimbursement and up to $50 for taxi or shuttle service to the study site

11.5. STUDY DISCONTINUATION

The study may be discontinued at any time by the investigators, the Emory IRB, the OHRP or other government agencies as part of their duties to ensure that research participants are protected.

12. PUBLICATION OF RESEARCH FINDINGS

Publication of the results of this trial will be governed by the policies and procedures of the NIA and Emory.

13. REFERENCES

1. Hajjar I, Hart M, Chen YL, et al. Antihypertensive therapy and cerebral hemodynamics in executive mild cognitive impairment: results of a pilot randomized clinical trial. *J Am Geriatr Soc.* 2013;61(2):194-201.
2. Kume K, Hanyu H, Sakurai H, Takada Y, Onuma T, Iwamoto T. Effects of telmisartan on cognition and regional cerebral blood flow in hypertensive patients with Alzheimer's disease. *Geriatrics & gerontology international.* 2012;12(2):207-214.
3. Zhang R, Bai YG, Lin LJ, et al. Blockade of AT1 receptor partially restores vasoreactivity, NOS expression, and superoxide levels in cerebral and carotid arteries of hindlimb unweighting rats. *Journal of applied physiology.* 2009;106(1):251-258.
4. Cantin S, Villien M, Moreaud O, et al. Impaired cerebral vasoreactivity to CO2 in Alzheimer's disease using BOLD fMRI. *Neuroimage.* 2011;58(2):579-587.
5. Hajjar I, Hart M, Mack W, Lipsitz LA. Aldosterone, Cognitive Function, and Cerebral Hemodynamics in Hypertension and Antihypertensive Therapy. *American journal of hypertension.* 2014.
6. Hughes TM, Kuller LH, Barinas-Mitchell EJ, et al. Arterial Stiffness and beta-Amyloid Progression in Nondemented Elderly Adults. *JAMA neurology.* 2014;71(5):562-568.
7. King KS. Arterial Stiffness as a Potential Determinant of beta-Amyloid Deposition. *JAMA neurology.* 2014;71(5):541-542.
8. Kong XD, Zhang Y, Liu L, Sun N, Zhang MY, Zhang JN. Endothelial progenitor cells with Alzheimer's disease. *Chin Med J (Engl).* 2011;124(6):901-906.
9. Walker PA, Shah SK, Jimenez F, et al. Intravenous multipotent adult progenitor cell therapy for traumatic brain injury: preserving the blood brain barrier via an interaction with splenocytes. *Experimental neurology.* 2010;225(2):341-352.
10. Lippmann ES, Al-Ahmad A, Palecek SP, Shusta EV. Modeling the blood-brain barrier using stem cell sources. *Fluids and barriers of the CNS.* 2013;10(1):2.
11. Ponio JB, El-Ayoubi F, Glacial F, et al. Instruction of Circulating Endothelial Progenitors In Vitro towards Specialized Blood-Brain Barrier and Arterial Phenotypes. *PLoS One.* 2014;9(1):e84179.
12. Lippmann ES, Azarin SM, Kay JE, et al. Derivation of blood-brain barrier endothelial cells from human pluripotent stem cells. *Nature biotechnology.* 2012;30(8):783-791.
13. Zhang ZG, Zhang L, Jiang Q, Chopp M. Bone marrow-derived endothelial progenitor cells participate in cerebral neovascularization after focal cerebral ischemia in the adult mouse. *Circulation research.* 2002;90(3):284-288.
14. Berk BC, Rao GN. Angiotensin II-induced vascular smooth muscle cell hypertrophy: PDGF A-chain mediates the increase in cell size. *Journal of cellular physiology.* 1993;154(2):368-380.
15. Suzuki Y, Ruiz-Ortega M, Lorenzo O, Ruperez M, Esteban V, Egido J. Inflammation and angiotensin II. *The international journal of biochemistry & cell biology.* 2003;35(6):881-900.
16. Park S, Lakatta EG. Role of inflammation in the pathogenesis of arterial stiffness. *Yonsei medical journal.* 2012;53(2):258-261.
17. Lee ST, Chu K, Jung KH, et al. Dysfunctional characteristics of circulating angiogenic cells in Alzheimer's disease. *Journal of Alzheimer's disease : JAD.* 2010;19(4):1231-1240.
18. Endtmann C, Ebrahimian T, Czech T, et al. Angiotensin II impairs endothelial progenitor cell number and function in vitro and in vivo: implications for vascular regeneration. *Hypertension.* 2011;58(3):394-403.
19. Suzuki R, Fukuda N, Katakawa M, et al. Effects of an angiotensin II receptor blocker on the impaired function of endothelial progenitor cells in patients with essential hypertension. *Am J Hypertens.* 2014;27(5):695-701.
20. Zhao W, Wang J, Ho L, Ono K, Teplow DB, Pasinetti GM. Identification of antihypertensive drugs which inhibit amyloid-beta protein oligomerization. *Journal of Alzheimer's disease : JAD.* 2009;16(1):49-57.
21. Wang J, Ho L, Chen L, et al. Valsartan lowers brain beta-amyloid protein levels and improves spatial learning in a mouse model of Alzheimer disease. *J Clin Invest.* 2007;117(11):3393-3402.
22. Zhu D, Shi J, Zhang Y, et al. Central angiotensin II stimulation promotes beta amyloid production in Sprague Dawley rats. *PLoS One.* 2011;6(1):e16037.
23. Deane R, Bell RD, Sagare A, Zlokovic BV. Clearance of amyloid-beta peptide across the blood-brain barrier: implication for therapies in Alzheimer's disease. *CNS & neurological disorders drug targets.* 2009;8(1):16-30.

24. Zhang M, Mao Y, Ramirez SH, Tuma RF, Chabrashvili T. Angiotensin II induced cerebral microvascular inflammation and increased blood-brain barrier permeability via oxidative stress. *Neuroscience.* 2010;171(3):852-858.
25. Ito S, Matsumiya K, Ohtsuki S, Kamiie J, Terasaki T. Contributions of degradation and brain-to-blood elimination across the blood-brain barrier to cerebral clearance of human amyloid-beta peptide(1-40) in mouse brain. *Journal of cerebral blood flow and metabolism : official journal of the International Society of Cerebral Blood Flow and Metabolism.* 2013;33(11):1770-1777.
26. Pflanzner T, Kuhlmann CR, Pietrzik CU. Blood-brain-barrier models for the investigation of transporter- and receptor-mediated amyloid-beta clearance in Alzheimer's disease. *Current Alzheimer research.* 2010;7(7):578-590.
27. Grossin N, Boulanger E, Wautier MP, Wautier JL. The different isoforms of the receptor for advanced glycation end products are modulated by pharmacological agents. *Clinical hemorheology and microcirculation.* 2010;45(2-4):143-153.
28. Agarwal D, Dange RB, Raizada MK, Francis J. Angiotensin II causes imbalance between pro- and anti-inflammatory cytokines by modulating GSK-3beta in neuronal culture. *Br J Pharmacol.* 2013;169(4):860-874.
29. Medina M, Garrido JJ, Wandosell FG. Modulation of GSK-3 as a Therapeutic Strategy on Tau Pathologies. *Frontiers in molecular neuroscience.* 2011;4:24.
30. Wang JZ, Wu Q, Smith A, Grundke-Iqbal I, Iqbal K. Tau is phosphorylated by GSK-3 at several sites found in Alzheimer disease and its biological activity markedly inhibited only after it is prephosphorylated by A-kinase. *FEBS letters.* 1998;436(1):28-34.
31. Forlenza OV, Torres CA, Talib LL, et al. Increased platelet GSK3B activity in patients with mild cognitive impairment and Alzheimer's disease. *Journal of psychiatric research.* 2011;45(2):220-224.
32. Tian M, Zhu D, Xie W, Shi J. Central angiotensin II-induced Alzheimer-like tau phosphorylation in normal rat brains. *FEBS letters.* 2012;586(20):3737-3745.
33. Zandbergen AAM, Baggen MGA, Lamberts SWJ, Bootsma AH, de Zeeuw D, Ouwendijk RJT. Effect of Losartan on Microalbuminuria in Normotensive Patients with Type 2 Diabetes MellitusA Randomized Clinical Trial. *Annals of Internal Medicine.* 2003;139(2):90-96.
34. Sjolie AK, Klein R, Porta M, et al. Effect of candesartan on progression and regression of retinopathy in type 2 diabetes (DIRECT-Protect 2): a randomised placebo-controlled trial. *Lancet.* 2008;372(9647):1385-1393.
35. Das UN. Is angiotensin-II an endogenous pro-inflammatory molecule? *Med Sci Monit.* 2005;11(5):RA155-162.
36. Kumaran D, Udayabanu M, Kumar M, Aneja R, Katyal A. Involvement of angiotensin converting enzyme in cerebral hypoperfusion induced anterograde memory impairment and cholinergic dysfunction in rats. *Neuroscience.* 2008;155(3):626-639.
37. Barnes JM, Barnes NM, Costall B, et al. Angiotensin II inhibits cortical cholinergic function: implications for cognition. *J Cardiovasc Pharmacol.* 1990;16(2):234-238.
38. Tota S, Goel R, Pachauri SD, et al. Effect of angiotensin II on spatial memory, cerebral blood flow, cholinergic neurotransmission, and brain derived neurotrophic factor in rats. *Psychopharmacology (Berl).* 2012.
39. Yonkov DI, Georgiev VP. Cholinergic influence on memory facilitation induced by angiotensin II in rats. *Neuropeptides.* 1990;16(3):157-162.
40. Iwai M, Liu HW, Chen R, et al. Possible inhibition of focal cerebral ischemia by angiotensin II type 2 receptor stimulation. *Circulation.* 2004;110(7):843-848.
41. Reinecke K, Lucius R, Reinecke A, Rickert U, Herdegen T, Unger T. Angiotensin II accelerates functional recovery in the rat sciatic nerve in vivo: role of the AT2 receptor and the transcription factor NF-kappaB. *Faseb J.* 2003;17(14):2094-2096.
42. Horiuchi M, Mogi M. Roles of Activation of Angiotensin II Receptor Subtypes in Ischemic Brain Damage and Cognitive Function. *Br J Pharmacol.* 2010.
43. Horiuchi M, Mogi M, Iwai M. The angiotensin II type 2 receptor in the brain. *J Renin Angiotensin Aldosterone Syst.* 2010;11(1):1-6.
44. Rompe F, Artuc M, Hallberg A, et al. Direct angiotensin II type 2 receptor stimulation acts anti-inflammatory through epoxyeicosatrienoic acid and inhibition of nuclear factor kappaB. *Hypertension.* 2010;55(4):924-931.
45. Wilms H, Rosenstiel P, Unger T, Deuschl G, Lucius R. Neuroprotection with angiotensin receptor antagonists: a review of the evidence and potential mechanisms. *American journal of cardiovascular drugs : drugs, devices, and other interventions.* 2005;5(4):245-253.

46. Grammatopoulos T, Morris K, Ferguson P, Weyhenmeyer J. Angiotensin protects cortical neurons from hypoxic-induced apoptosis via the angiotensin type 2 receptor. *Brain Res Mol Brain Res.* 2002;99(2):114-124.
47. Jiang T, Gao L, Shi J, Lu J, Wang Y, Zhang Y. Angiotensin-(1-7) modulates renin-angiotensin system associated with reducing oxidative stress and attenuating neuronal apoptosis in the brain of hypertensive rats. *Pharmacol Res.* 2013;67(1):84-93.
48. Shenoy UV, Richards EM, Huang XC, Sumners C. Angiotensin II type 2 receptor-mediated apoptosis of cultured neurons from newborn rat brain. *Endocrinology.* 1999;140(1):500-509.
49. Steckelings UM, Kaschina E, Unger T. The AT2 receptor--a matter of love and hate. *Peptides.* 2005;26(8):1401-1409.
50. Wright JW, Reichert JR, Davis CJ, Harding JW. Neural plasticity and the brain renin-angiotensin system. *Neurosci Biobehav Rev.* 2002;26(5):529-552.
51. Hajjar I, Rodgers K. Do angiotensin receptor blockers prevent Alzheimer's disease? *Curr Opin Cardiol.* 2013;28(4):417-425.
52. Lawton MP, Brody EM. Assessment of older people: self-maintaining and instrumental activities of daily living. *The Gerontologist.* 1969;9(3):179-186.
53. Guralnik JM, Simonsick EM, Ferrucci L, et al. A short physical performance battery assessing lower extremity function: association with self-reported disability and prediction of mortality and nursing home admission. *J Gerontol.* 1994;49(2):M85-94.
54. Kramer JH. Special series introduction: NIH EXAMINER and the assessment of executive functioning. *Journal of the International Neuropsychological Society : JINS.* 2014;20(1):8-10.
55. Eriksen B, Eriksen C. Effects of noise letters upon the identification of a target letter in a nonsearch task. *Attention, Perception, & Psychophysics.* 1974;16(1):143-149.
56. Lowe C, Rabbitt P. Test/re-test reliability of the CANTAB and ISPOCD neuropsychological batteries: theoretical and practical issues. Cambridge Neuropsychological Test Automated Battery. International Study of Post-Operative Cognitive Dysfunction. *Neuropsychologia.* 1998;36(9):915-923.
57. Owen AM, Downes JJ, Sahakian BJ, Polkey CE, Robbins TW. Planning and spatial working memory following frontal lobe lesions in man. *Neuropsychologia.* 1990;28(10):1021-1034.
58. Ross TP. The reliability of cluster and switch scores for the Controlled Oral Word Association Test. *Arch Clin Neuropsychol.* 2003;18(2):153-164.
59. Acevedo A, Krueger KR, Navarro E, et al. The Spanish translation and adaptation of the Uniform Data Set of the National Institute on Aging Alzheimer's Disease Centers. *Alzheimer Dis Assoc Disord.* 2009;23(2):102-109.
60. Weintraub S, Salmon D, Mercaldo N, et al. The Alzheimer's Disease Centers' Uniform Data Set (UDS): the neuropsychologic test battery. *Alzheimer Dis Assoc Disord.* 2009;23(2):91-101.
61. Delis DC, Freeland J, Kramer JH, Kaplan E. Integrating clinical assessment with cognitive neuroscience: construct validation of the California Verbal Learning Test. *J Consult Clin Psychol.* 1988;56(1):123-130.
62. Kreiner DS, Ryan JJ. Memory and motor skill components of the WAIS-III Digit Symbol-Coding subtest. *Clin Neuropsychol.* 2001;15(1):109-113.
63. Wechsler D. *WMS-R : Wechsler Memory Scale--Revised : manual.* San Antonio: Psychological Corp. : Harcourt Brace Jovanovich; 1987.
64. Mack WJ, Freed DM, Williams BW, Henderson VW. Boston Naming Test: shortened versions for use in Alzheimer's disease. *J Gerontol.* 1992;47(3):P154-158.
65. Canning SJ, Leach L, Stuss D, Ngo L, Black SE. Diagnostic utility of abbreviated fluency measures in Alzheimer disease and vascular dementia. *Neurology.* 2004;62(4):556-562.
66. Radloff LS. The CES-D Scale: A Self-Report Depression Scale for Research in the General Population. *Applied Psychological Measurement.* 1977;1(3):385-401.
67. Peskind ER, Riekse R, Quinn JF, et al. Safety and acceptability of the research lumbar puncture. *Alzheimer disease and associated disorders.* 2005;19(4):220-225.
68. Olsson A, Vanderstichele H, Andreasen N, et al. Simultaneous measurement of beta-amyloid(1-42), total tau, and phosphorylated tau (Thr181) in cerebrospinal fluid by the xMAP technology. *Clinical chemistry.* 2005;51(2):336-345.
69. Nitkunan A, Barrick TR, Charlton RA, Clark CA, Markus HS. Multimodal MRI in cerebral small vessel disease: its relationship with cognition and sensitivity to change over time. *Stroke; a journal of cerebral circulation.* 2008;39(7):1999-2005.

70. Lowe MJ. A historical perspective on the evolution of resting-state functional connectivity with MRI. *MAGMA.*23(5-6):279-288.
71. Klunk WE, Engler H, Nordberg A, et al. Imaging brain amyloid in Alzheimer's disease with Pittsburgh Compound-B. *Annals of neurology.* 2004;55(3):306-319.
72. Jagust WJ, Bandy D, Chen K, et al. The Alzheimer's Disease Neuroimaging Initiative positron emission tomography core. *Alzheimer's & dementia : the journal of the Alzheimer's Association.* 2010;6(3):221-229.
73. Klunk WE, Koeppe RA, Price JC, et al. The Centiloid Project: Standardizing quantitative amyloid plaque estimation by PET. *Alzheimer's & dementia : the journal of the Alzheimer's Association.* 2015;11(1):1-15 e14.
74. Chien DT, Bahri S, Szardenings AK, et al. Early clinical PET imaging results with the novel PHF-tau radioligand [F-18]-T807. *Journal of Alzheimer's disease : JAD.* 2013;34(2):457-468.
75. Okamura N, Harada R, Furumoto S, Arai H, Yanai K, Kudo Y. Tau PET imaging in Alzheimer's disease. *Current neurology and neuroscience reports.* 2014;14(11):500.
76. Shoup TM, Yokell DL, Rice PA, et al. A concise radiosynthesis of the tau radiopharmaceutical, [(18) F]T807. *Journal of labelled compounds & radiopharmaceuticals.* 2013;56(14):736-740.
77. Xia CF, Arteaga J, Chen G, et al. [(18)F]T807, a novel tau positron emission tomography imaging agent for Alzheimer's disease. *Alzheimer's & dementia : the journal of the Alzheimer's Association.* 2013;9(6):666-676.
78. Zimmer ER, Leuzy A, Gauthier S, Rosa-Neto P. Developments in Tau PET Imaging. *The Canadian journal of neurological sciences. Le journal canadien des sciences neurologiques.* 2014;41(5):547-553.
79. Hill JM, Zalos G, Halcox JP, et al. Circulating endothelial progenitor cells, vascular function, and cardiovascular risk. *The New England journal of medicine.* 2003;348(7):593-600.
80. Fadini GP, Baesso I, Albiero M, Sartore S, Agostini C, Avogaro A. Technical notes on endothelial progenitor cells: ways to escape from the knowledge plateau. *Atherosclerosis.* 2008;197(2):496-503.
81. Vasa M, Fichtlscherer S, Aicher A, et al. Number and migratory activity of circulating endothelial progenitor cells inversely correlate with risk factors for coronary artery disease. *Circ Res.* 2001;89(1):E1-7.
82. Surinova S, Huttenhain R, Chang CY, Espona L, Vitek O, Aebersold R. Automated selected reaction monitoring data analysis workflow for large-scale targeted proteomic studies. *Nature protocols.* 2013;8(8):1602-1619.
83. Ewers M, Mielke MM, Hampel H. Blood-based biomarkers of microvascular pathology in Alzheimer's disease. *Experimental gerontology.* 2010;45(1):75-79.
84. Hu WT, Holtzman DM, Fagan AM, et al. Plasma multianalyte profiling in mild cognitive impairment and Alzheimer disease. *Neurology.* 2012;79(9):897-905.
85. Casadesus G, Smith MA, Basu S, et al. Increased isoprostane and prostaglandin are prominent in neurons in Alzheimer disease. *Molecular neurodegeneration.* 2007;2:2.
86. Montine TJ, Kaye JA, Montine KS, McFarland L, Morrow JD, Quinn JF. Cerebrospinal fluid abeta42, tau, and f2-isoprostane concentrations in patients with Alzheimer disease, other dementias, and in age-matched controls. *Archives of pathology & laboratory medicine.* 2001;125(4):510-512.
87. Schmidt FM, Mergl R, Stach B, Jahn I, Gertz HJ, Schonknecht P. Elevated levels of cerebrospinal fluid neuron-specific enolase (NSE) in Alzheimer's disease. *Neuroscience letters.* 2014;570:81-85.
88. Steiner J, Bogerts B, Schroeter ML, Bernstein HG. S100B protein in neurodegenerative disorders. *Clinical chemistry and laboratory medicine : CCLM / FESCC.* 2011;49(3):409-424.
89. Deane R, Wu Z, Zlokovic BV. RAGE (yin) versus LRP (yang) balance regulates alzheimer amyloid beta-peptide clearance through transport across the blood-brain barrier. *Stroke.* 2004;35(11 Suppl 1):2628-2631.
90. Ye M, Wysocki J, Gonzalez-Pacheco FR, et al. Murine recombinant angiotensin-converting enzyme 2: effect on angiotensin II-dependent hypertension and distinctive angiotensin-converting enzyme 2 inhibitor characteristics on rodent and human angiotensin-converting enzyme 2. *Hypertension.* 2012;60(3):730-740.
91. Jeste DV, Palmer BW, Appelbaum PS, et al. A new brief instrument for assessing decisional capacity for clinical research. *Arch Gen Psychiatry.* 2007;64(8):966-974.
92. Appelbaum PS, Grisso T. *MacArthur competence assessment tool for clinical research (MacCAT-CR).* Sarasota, FL: Professional Resource Press; 2001.
93. Stroup S, Appelbaum P, Swartz M, et al. Decision-making capacity for research participation among individuals in the CATIE schizophrenia trial. *Schizophr Res.* 2005;80(1):1-8.

94. Pickering TG, Hall JE, Appel LJ, Falkner BE, Graves J, Hill MN, Jones DW, Kurtz T, Sheps SG, Roccella EJ. Recommendations for blood pressure measurement in humans and experimental animals: Part 1: Blood pressure measurement in humans: A statement for professionals from the subcommittee of professional and public education of the american heart association council on high blood pressure research. Hypertension. 2005;45:142-161.
95. Ashfaq S, Abramson JL, Jones DP, Rhodes SD, Weintraub WS, Hooper WC, Vaccarino V, Harrison DG, Quyyumi AA. The relationship between plasma levels of oxidized and reduced thiols and early atherosclerosis in healthy adults. Journal of the American College of Cardiology. 2006;47:1005-1011.

Candesartan’s Effects on Alzheimer’s Disease and Related Biomarkers

(CEDAR)

STATISTICAL ANALYSIS PLAN

Maureen Okafor, MD MPH
Reneé Moore, PhD
Ihab Hajjar, MD

Emory University School of Medicine

TABLE OF CONTENTS

1. BACKGROUND AND RATIONALE……………….……………..……..…………………..ERROR! BOOKMARK NOT DEFINED.

2. STUDY OBJECTIVES……………………………………………………………………..ERROR! BOOKMARK NOT DEFINED.

2.1. Aim 1…………………………………………………………………………………**ERROR! BOOKMARK NOT DEFINED.**

2.2. Aim 2…………………………………………………………………………………**ERROR! BOOKMARK NOT DEFINED.**

3. SELECTION AND ENROLLMENT……………………………………………………...4

3.1. Inclusion Criteria……………………………………………………………...…4

3.2. Exclusion Criteria…………………………………….…………………………4

4. POWER AND SAMPLE SIZE…………………………………………………………..5

5. DATA ANALYSIS AND STATISTICS…...……………………………………………..5

5.1. Outcomes………………………………..…………………………………………5

5.2. Statistical Procedures ………………….…..……………………………………………6

5.3. Outcome Analysis……………………………………………………………..…..……...6

5.4. Missing Data and Statistical Models…………………………………………………..7

5.5 Sensitivity Analysis and Dose Response………………………………………………7

5.6 Interim Analysis………………………………………..……………...……….…....8

6. QUALITY ASSURANCE PLANS……………………………………………………….8

7. PROGRAMMING PLANS……………………………………………………………….8

8. TABLES AND FIGURES………………………………………………………………….8

8.1. Tables……………………………………………………………………………..8

8.2. Figures……………………………………………………………………………8

1. BACKGROUND AND RATIONALE

The renin angiotensin aldosterone system (RAAS) is an ideal system to target for safe and effective pharmacological modulation of vascular factors centrally involved in Alzheimer's disease (AD) pathogenesis (Kehoe et al, 2003). Specifically, angiotensin receptor blockers (ARBs) may positively alter many indicators that lead to increased amyloid deposition and vascular pathology in the brain such as vascular stiffness and inflammation, cerebral perfusion, and endothelial dysfunction. Results from more than 600 brain autopsies show that those who were treated with ARBs pre-mortem had significantly lower markers of amyloid deposition and to a greater extent, lower tau-based neurofibrillary tangles in the brain. The multi-target characteristic of ARBs increases the likelihood of producing a disease-modifying effect, rather than a symptomatic effect as provided by currently approved medications. See trial protocol for more detail.

2. STUDY OBJECTIVES

Available treatments for Alzheimer's disease do not address novel neurovascular and inflammatory pathways. Prior research has documented that antihypertensive angiotensin receptor blockers (ARBs), in general, and candesartan, in particular, may have a positive effect on both AD biomarkers and clinical manifestations. We hypothesize that by blocking the effect of angiotensin II, a crucial mediator of neurovascular injury, candesartan may address the effect of vascular function, blood pressure (BP), inflammation, and the amyloid cycle on Alzheimer's disease.

Our *primary objective* is to determine the safety and dose-response for target engagement of the treatment with escalating doses of candesartan in those with mild cognitive impairment (MCI) and AD based on cerebrospinal fluid (CSF) levels of amyloid and tau.

Our *secondary objectives* are to assess the efficacy of 1-year treatment of candesartan on biomarkers of vascular function, neurodegeneration, and neuro-inflammation, to assess the efficacy of 1-year treatment with candesartan on amyloid imaging, and to estimate the effect size of candesartan on cognitive and functional measures in MCI and AD.

2.1. Aim 1

To determine the safety and tolerability of treatment with escalating doses of candesartan. *Hypotheses:* In non-hypertensive individuals with MCI-AD, treatment with candesartan at maximal tolerable dose is safe and is associated with hemodynamic (symptomatic hypotension) and non-hemodynamic (hyperkalemia and increased serum creatinine) events comparable to placebo.

2.2. Aim 2

To assess the efficacy of candesartan on AD-related neurovascular biomarkers.
Hypotheses: In non-hypertensive individuals with MCI-AD, candesartan is associated with detectable and significant declines in CSF tau, fibrillary amyloid-β load on positron emission tomography (PET) imaging, inflammatory markers, increased cerebral perfusion (arterial spin labeling MRI), and improved markers of vascular function relative to placebo.

As a secondary goal of this phase II study, we aim to further investigate the effect of candesartan on clinical outcomes and tau PET imaging in MCI-AD. We hypothesize that there will be a detectable positive impact of candesartan on memory and executive function and on tau PET uptake in MCI-AD. This will provide power estimates for a future phase III trial.

3. SELECTION AND ENROLLMENT

3.1. Inclusion Criteria

(1) Age: 50 years or older

(2) MCI will be defined as:

(i) Subjective memory concern

(ii) Abnormal memory function documented using the Logical Memory subscale (Delayed Paragraph Recall, Paragraph A only) from the Wechsler Memory Scale-Revised (the maximum score is 25): [< 11 for 16 or more years of education; < 9 for 8-15 years of education; < 6 for < 7 years of education];

(iii) Montreal Cognitive Assessment (MoCA) < 26

(iv) Clinical Dementia Rating scale /Memory sum Box score = 0.5

(v) General functional performance sufficiently preserved (Functional Assessment Questionnaire < 9)

(3) Amyloid positivity determined by measuring the amyloid content in the brain assessed by either CSF amyloid level or an amyloid scan (^{11}C-PiB PET). We plan to use CSF levels of amyloid as our default method to assess amyloid positivity. However, if we are unable to determine eligibility via a lumbar puncture procedure, then a PiB PET will be performed.

In instances where the diagnosis of MCI is not clear, a review of the participant's cognitive and non-cognitive data will be performed by the study physicians or neuropsychologists and a diagnosis of MCI will be based on the clinical judgment of the study physicians or neuropsychologists.

3.2. Exclusion Criteria

(1) Intolerance to ARBs

(2) Current use of ARBs, angiotensin-converting enzyme inhibitors (ACEIs) (the use of antihypertensive medications other than ACEI or ARBs for other indications is allowed)

(3) Current diagnosis of hypertension or current use of antihypertensive medication that is prescribed specifically for hypertensive therapy

(4) SBP less than 110 or DBP less than 40 mm Hg

(5) Renal disease (serum creatinine >2.0 mg/dl), hyperkalemia (serum potassium >5.5 mEq/dl), platelets < 50,000/µl, or international normalized ratio (INR) >1.9

(6) Active medical or psychiatric diseases that in the judgment of the investigator would affect the safety of the participant or scientific integrity of the study

(7) Uncontrolled congestive heart failure reflected by poor exercise tolerance and shortness of breath

(8) History of stroke in the past 3 years

(9) Inability to have MRI (e.g. metal implants or cardiac pacemaker) with an exception for those who cannot have an MRI (if all other parts of the study are obtained successfully, they may still be enrolled in the study), or inability to perform cognitive assessment or to assess amyloid positivity (no LP and no amyloid scan);

(10) History of increased intracranial pressure (ICP) or bleeding diathesis (from disease states or from use of anticoagulants such as warfarin, heparin and related products, rivaroxaban or Xarelto, apixaban or Eliquis, edoxaban or Savaysa, dabigatran or Pradaxa)

(11) Women of childbearing potential (non-menopausal)

(12) In those who are unable to demonstrate that they understood the details of the study (i.e. lack of decisional capacity to consent), a study partner/surrogate who can consent on their behalf will be required, otherwise, they will be excluded

(13) Current use of Lithium, as candesartan may increase lithium concentration to toxic levels.

4. POWER AND SAMPLE SIZE

Based on a preliminary analysis of ADNI data, the effect size of ARBs on CSF total tau = 6, phosphorylated tau = 6.6 and on amyloid PET = 0.1. We performed our power analysis to detect these effects. At an $\alpha = 0.05$ and $\beta = 0.8$, a sample of 20 per group (total sample of 40) was estimated to provide power to detect an effect ≥ 5.6 for total tau and p-tau. This sample size would also allow us to detect an effect 0.45 for PWV and 0.27 for PC.16 For the amyloid PET scanning, a sample size of 10 per group would allow a detectable effect of ≥ 0.1. Both our ADNI analysis and the yearly change in the placebo arm of the bapineuzumab trial (Rinne O.J. et al., 2010) suggested that the yearly change in ^{11}C-PiB SUVr is in the neighborhood of 0.2 (SD = 0.08). There are no data available about the rate of change in ^{18}F-T807 SUVr. Based on previous studies, the SD of global SUVr for T807 is 0.09. Hence, we would be able to detect the same effect for ^{18}F-T807 as for ^{11}C-PiB PET. The trial has sufficient resources to enroll 72 participants who will be randomly allocated in equal proportions to each of the 2 groups – candesartan and placebo. All 72 subjects will be invited to participate in the additional PET study.

5. DATA ANALYSIS AND STATISTICS

5.1. Outcomes

Primary Outcome Measures: The primary outcome for this study is safety of using candesartan in non-hypertensive individuals with MCI. Safety will be measured by: the number of hypotensive episodes (defined as blood pressure <100/40 mm Hg averaged at each visit), the number of subjects with symptoms of hypotension such as dizziness, weakness or fatigue, number of participants with serum creatinine >2.5 mg/dl, number of participants with serum potassium >5.9 mEq/dl, and number of discontinuations of candesartan due to any reason. All randomized participants will be included in the safety analysis.

Secondary Outcome Measures: The secondary outcomes include: CSF tau (both total and p-tau), CSF inflammatory proteins, global PiB and T807 standardized uptake value ratio (SUVr), neuroimaging measures (global cerebral perfusion, CO_2 vasoreactivity, and hippocampal volume), pulse wave velocity (PWV), and augmentation index.

Exploratory and other prespecified Outcome: global cognitive and functional measure (using Clinical Dementia Rating scale), executive function (using Trail Making Test Parts B and B-A and EXAMINER tool) and memory (using HVLT-R).

5.2. Statistical Procedures

Baseline characteristics including demographic, APOe, social and cognitive measures will be compared between the 2 groups to assess randomization fidelity. Univariate summary statistics will be provided for continuous variables and frequencies for categorical variables. Graphical methods will be used to examine distributions and identify potential influential points. The balance of baseline measures across the two groups will be compared using appropriate 2-sample tests, including Wilcoxon rank-sum and t-tests, Fisher's exact and Chi-square tests as appropriate.

5.3. Outcome Analysis

All analyses will be conducted using the intention-to-treat (ITT) principle in which all available data on all randomized participants are included. The primary outcome analysis will utilize the Chi-square test to compare safety measures between the candesartan and placebo groups. Secondary outcome analysis will utilize mixed models with repeated measures (MMRM) with unstructured covariance to compare CSF biomarkers, vascular and neuroimaging outcomes between the candesartan and placebo groups. Our main explanatory variable is the indicator for treatment group.

The test of treatment effects on trial outcomes will be an interaction of treatment by visit. The main covariate adjustments will be stratification variable (use of cholinesterase inhibitors or memantine). Summary statistics will include the model-adjusted mean (LSM) and 95% confidence interval for each treatment group at each visit, and the mean (95% confidence interval) group difference on each outcome over the 12 months (effect size). All tests of significance on treatment group comparisons will use a two-sided alpha of 0.05. The inflammatory markers data analyses will look for an association between the change in protein measures (increase or decrease) and candesartan for each protein in the participant targeted proteins.

5.4. Missing data and statistical models

Because all analyses will be conducted using the intention-to-treat (ITT) principle, this approach minimizes bias if individuals drop out of the two arms for different reasons. Every effort will be made to obtain follow-up data on all participants randomized, whether or not they follow their assigned treatment. Our maximum likelihood approach assumes that any missing outcome data is missing at random, i.e. missing data including that due to drop-out can be dependent on any previously observed outcomes or

treatment assignment (*Little RJA. Modeling the dropout mechanism in repeated-measures studies. J Am Statist. Assn. 1993; 88:125-34*). With this approach, we use all data that have been collected without regard to whether data are missing for a patient at another visit, including drop-out, and without explicit imputation of missing data.

5.5. Sensitivity Analysis and Dose Response

For outcome measures that differed at baseline between the 2 groups, a sensitivity analysis will be conducted by adjusting the baseline measure for that outcome. For those instances, we will provide unadjusted and adjusted results. We will further assess dose response for target engagement by comparing randomization to low dose vs. high dose of candesartan.

5.6. Interim Analysis

Due to the sample size of this study, no interim analysis is planned for this project.

6. QUALITY ASSURANCE PLANS

To assess quality of data collected, the PI along with another investigator will randomly and on intervals review data obtained. In addition, yearly assessment of personnel competencies in obtaining data (e.g. blood pressure checks, neuropsychological assessments, etc.) will be performed. Data entry into the trial's REDCap database will be checked by a second operator to match the source documents with the database.

7. PROGRAMMING PLANS

SAS 9.4 will be used for all analyses by study statisticians. For mixed models, the below SAS code structure example will be used:

```
proc mixed data=dataset covtest;
class id   visit(ref='1') treatment;
model outcome = visit treatment visit* treatment /s cl;
repeated  / type=un subject=id;
lsmeans visit* treatment /pdiff cl;
quit;
```

8. TABLES AND FIGURES

8.1. Tables

(1) Demographic Characteristics
(2) Adverse Events

(3) Complete vs. Dropout
(4) Outcomes Linear Mixed Method results

8.2. Figures

(1) Consort Diagram
(2) Potassium, Creatinine and Blood Pressure
(3) Cognitive Outcomes
(4) CSF Outcomes
(5) Vascular Outcomes
(6) neuroimaging Outcomes
